# Uncertainty aware resilience enhancement of energy hub integrated distribution networks under heterogeneous disturbance events

**DOI:** 10.1016/j.isci.2026.117529

**Published:** 2026-09-15

**Authors:** Shahram Emaratkar, Majid Moradlou, Peyman Nazarian

**Affiliations:** 1 Department of Electrical Engineering, Za.C, Islamic Azad University, Zanjan, Iran

**Keywords:** energy hub-integrated distribution network, resilient operation, heterogeneous outage events, budgeted polyhedral adverse-point scheduling, network reconfiguration, energy storage system

## Abstract

This study develops a 24 h mixed integer linear programming framework for resilient operation of an integrated electricity, gas, and heat distribution system with energy hubs under prescribed outages and operating uncertainty. Electrical and thermal demand and wind and photovoltaic shortfalls are represented through budgeted polyhedral adverse point scheduling. Five cases compare a no-event reference, a combined outage baseline, radial reconfiguration, energy storage, and coordinated electrical and thermal demand response on the IEEE 33-bus system. Energy storage provides the strongest overall performance, reducing operating cost, expected energy not supplied, and peak shedding by 20.33%, 49.26%, and 30.44% relative to the combined outage baseline. Reconfiguration reduces shedding hours from 19 to 9 and the longest interruption from 19 to 5 h. Sensitivity and fixed plan stress analyses preserve the favorable storage ranking, showing that coordinated multi-energy flexibility can improve service continuity under heterogeneous disturbances.

## Highlights

• Energy storage reduces EENS by 49.26% under the combined disturbance
• Reconfiguration cuts shedding hours from 19 to 9 and longest interruption to 5 h
• Demand response reduces peak shedding by 18.20% without adding supply energy
• Stress analyses preserve the favorable ranking of the energy storage case

## Graphical abstract

**Figure:**
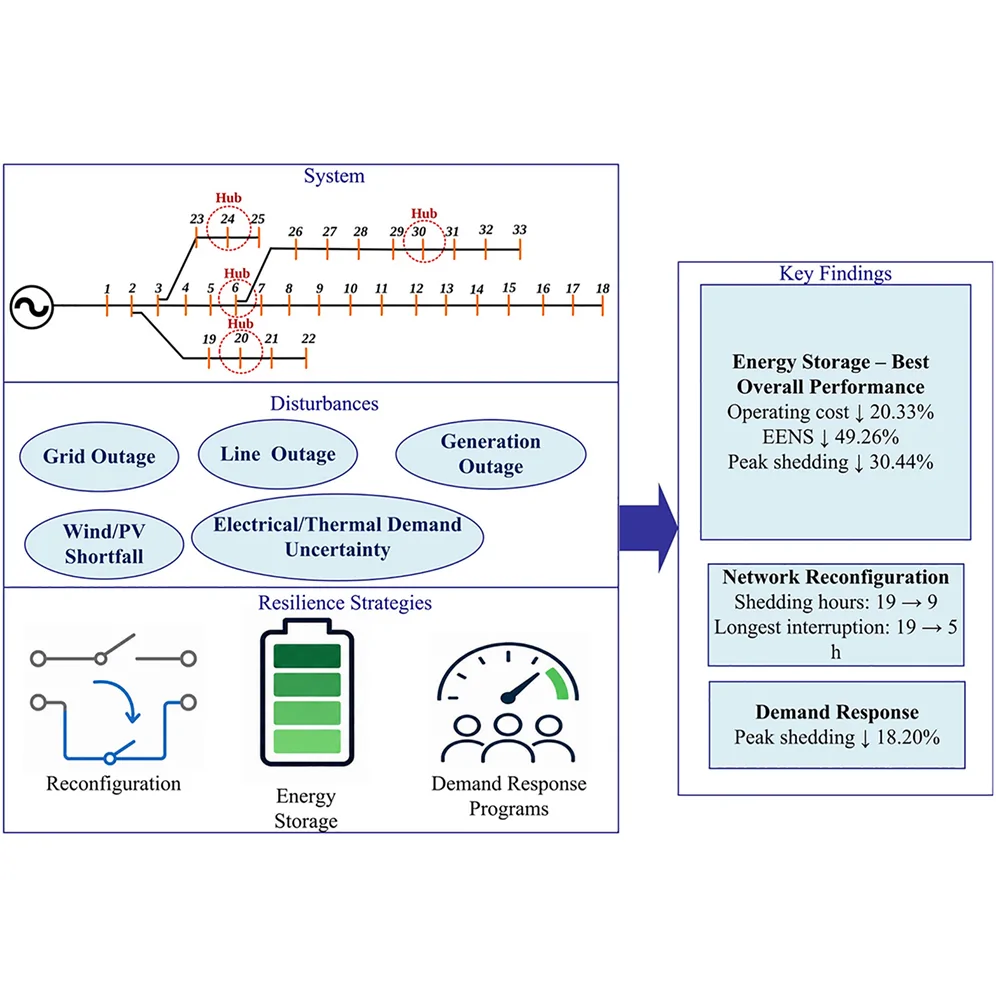

## Teaser

Applied sciences; Energy systems; Energy management

## Introduction

### Literature review

In ref.,^1^ the authors aimed to improve distribution network resilience under high-impact, low-probability events. Energy Storage Systems (ESS) and demand response were integrated into the operation model, while load and renewable generation uncertainties were handled using robust optimization. The main contributions include a resilience index and the coordinated assessment of flexible resources under different outage conditions on the IEEE 33-bus system. In ref.,^2^ the aim is to improve the Resilience Index (RI) of distribution networks against cyber-attacks using ESS. This paper uses a classical Robust Optimization (RO) method to manage the uncertainty of renewable energy sources and demand loads. In ref.,^3^ the aim is to enhance distribution network resilience under extreme natural disasters through a risk-oriented coordination framework for multiple virtual power plants within a reconfigurable network. This paper uses a bi-level optimization model transformed via Karush-Kuhn-Tucker (KKT) conditions into a single-level formulation, solved with GUROBI, while addressing uncertainty through Information Gap Decision Theory (IGDT) for both risk-averse and risk-seeking strategies. In ref.,^4^ the aim is to enhance the resilience of integrated power-gas-transportation systems against natural disasters by using energy hubs and multi-carrier mobile ESS for operational recovery of interrupted loads. This paper uses a resilience strategy implemented on a coupled 33-bus distribution network, 20-node gas network, and transportation network to manage the flexibility and mobility of energy carriers without relying on predefined uncertainty distributions. In,^5^ the objective of the study is to develop an efficient microgrid formation framework for resilient operation of hybrid AC/DC distribution systems, enabling flexible interconnection and coordinated scheduling of AC and DC networks to maximize critical load restoration under disruptive events. In,^6^ the objective of this study is to develop a resilient long-term planning framework for mobile ESS that accounts for renewable generation uncertainty, random failures, and operational complexity, by jointly optimizing investment decisions and routine and contingency-based grid operations to enhance distribution system resilience. In ref.^7^, the aim is to solve the joint planning-operation problem of resilient microgrid reconfiguration under uncertainty and ESS degradation, addressing both risk and technology-specific time-dependent losses. This paper uses a two-stage stochastic programming framework with conditional value-at-risk (CVaR) applied to second-stage operational costs, combined with a divide-and-conquer solution procedure, to hedge against extreme demand scenarios while handling capacity, reconfiguration, and degradation dynamics. In,^8^ the objective of the study is to develop a resilient distributed optimization framework for the economic dispatch of multi-energy systems that can maintain convergence and operational efficiency under denial-of-service attacks and constrained communication conditions. Faghihi Rezaei and Ghobadpour^9^ use reconfiguration to enhance the resilience of a real distribution network. Also, the work,^10^ examines enhancing distribution system resilience against extreme natural disasters using ESS. A two-stage robust optimization model is proposed, which first assesses component failure rates under the impact of secondary hazards and constructs a time-varying matrix of distribution line failures. The model aims to minimize the comprehensive economic cost of the ESS and the annual comprehensive weighted load loss, solved using the column-and-constraint generation algorithm. The goal of,^11^ is also to plan the resilience of the distribution system using storage systems and simultaneous combined heat and power (CHP) unit. Overall, this article aims to define an objective function to minimize the total investment costs in distributed generation, storage, and connection switches for reconfiguring the structure of microgrids to enhance losses and improve the reliability of distribution system consumers. Also,^12^ aims to improve distribution system operational efficiency and resilience by reducing power losses and enhancing voltage stability. To achieve this, the proposed model manages real-time operations and network reconfiguration using a robust optimization framework and a two-stage second-order cone optimization model. In work,^13^ a risk-averse stochastic mixed-integer programming model is proposed to facilitate cost-effective and resilient planning of hydrogen-integrated power distribution networks incorporating mobile hydrogen energy resources. The proposed planning framework follows a two-stage structure. In the first stage, the objective is to reduce investment costs through optimal siting and configuration of both stationary flexible energy resources and MHERs mobile hydrogen energy resources. In work,^14^ a proactive day-ahead scheduling strategy is investigated for distribution networks highly penetrated by renewable energy resources. The proposed method adopts a probabilistic scenario generation technique to model uncertainties associated with wind speed, solar radiation, disaster duration, and load variations. To address these uncertainties effectively, a hybrid stochastic p-robust optimization framework is applied, aiming to enhance decision-making and develop risk-resilient operational plans. In work,^15^ a seismic-resilient IES design model is proposed using a bi-level Monte Carlo method to capture low-probability seismic risks and optimize system configuration and operation under uncertainty. In work,^16^ a two-stage post-disaster repair and restoration strategy for distribution systems is developed. The approach co-optimizes repair crew scheduling, network reconfiguration, and DER dispatch to minimize operating costs under forecasted and real-time load conditions. In ref.,^17^ a bi-objective model is proposed to minimize operational cost and enhance resilience by reducing grid dependency and losses while maximizing hydrogen storage. A hybrid solution method combining goal programming and Generalized Benders Decomposition is used to solve the mixed-integer nonlinear problem. In,^18^ the authors propose a practical two-stage optimization framework to strengthen the resilience of power distribution systems following earthquakes. By using real seismic data and modeling uncertainties such as line failures, renewable variability, and electric vehicle behavior, the approach forms microgrids that restore more loads and significantly boost system resilience. In,^19^ the authors propose a flexible and adaptive scheduling approach to help microgrids stay resilient during extreme events. By combining unit commitment, network reconfiguration, and DR in a single model and accounting for uncertainties in renewables, load, and electricity prices the framework supports smarter, more reliable decision-making. The use of the pelican optimization algorithm ensures efficient operation, while the results highlight major improvements in microgrid performance and stability. In work,^20^ a robust operation model is developed to enhance the resilience of multi-carrier energy systems by leveraging the coordination between thermal and electrical sectors under high-risk conditions such as extreme weather events. Using IGDT to address power outage uncertainty, the proposed approach enables effective energy scheduling particularly through the flexible operation of CHP units thereby improving system performance, supporting successful islanding, and strengthening overall grid resilience. In ref.^21^, the aim is to minimize load shedding in distribution systems after severe disasters by offering incentive-oriented rewards to consumers through an incentive-based DR with optimal pricing based on distributed generation costs. This paper uses a Stackelberg game theory approach to determine day-ahead optimal incentive prices from the perspective of both consumers and distribution network operators, combined with machine learning for load forecasting and convexified formulations solved in GAMS.

Also, Wang et al.^22^ developed a tri-layer smart-switch deployment model that coordinates proactive islanding, fault propagation, isolation, and service restoration under line, substation-transformer, and switch failures. Critical scenarios were selected using a two-stage K-medoids procedure, and the model was reformulated as a scenario-based two-stage distributionally robust problem. Du and Du^23^ proposed a two-stage robust emergency scheduling framework that coordinates repair crews and emergency resources under the most-likely and most-severe multi-type fault scenarios; wind and photovoltaic uncertainty was represented by a Gaussian mixture model and the problem was solved using column-and-constraint generation. Liu et al.^24^ formulated a road-power-coupled multi-point fault repair and restoration problem, incorporated network reconfiguration, and developed an improved genetic algorithm using a comparison crossover operator and information-entropy-based population-diversity control.

Table 1 compares the reviewed studies in terms of system scope, heterogeneous outage representation, uncertainty treatment, flexibility mechanisms, recovery metrics, and Out-Of-Sample (OOS) validation. The comparison highlights the broader integration of these features within the proposed framework.

**Table 1:** Comparative assessment of the reviewed studies and the proposed framework

References	Resilience-oriented framework	Multi-energy distribution network	Heterogeneous outages	Uncertainty	Budgeted polyhedral adverse-point scheduling		Flexibility mechanisms			Assessment depth	
						Network reconfiguration	ESS	Electrical DR	Thermal DR	Recovery metrics	OOS validation
Huang et al.^1^	**✓**	✗	✗	**✓**	**✓**	✗	**✓**	**✓**	✗	✗	✗
Ghanbari-Ghalehjoughi et al.^2^	**✓**	✗	✗	**✓**	✗	✗	**✓**	✗	✗	✗	✗
Gholami et al.^3^	**✓**	✗	✗	**✓**	✗	**✓**	**✓**	**✓**	✗	**✓**	✗
Mohammadian-Alirezachaei et al.^4^	**✓**	**✓**	✗	**✓**	✗	✗	**✓**	✗	✗	**✓**	✗
Cai et al.^5^	**✓**	✗	✗	✗	✗	✗	**✓**	✗	✗	✗	✗
Dong et al.^6^	**✓**	✗	✗	**✓**	✗	**✓**	**✓**	✗	✗	✗	✗
Asghari et al.^7^	**✓**	✗	✗	**✓**	✗	**✓**	**✓**	✗	✗	✗	✗
Du et al.^8^	**✓**	**✓**	✗	**✓**	✗	✗	**✓**	✗	✗	✗	✗
Rezaei and Ghobadpour^9^	**✓**	✗	✗	**✓**	✗	**✓**	**✓**	✗	✗	✗	✗
He et al.^10^	**✓**	✗	**✓**	**✓**	✗	✗	**✓**	✗	✗	**✓**	✗
Khanavandi et al.^11^	**✓**	✗	✗	**✓**	✗	✗	**✓**	**✓**	✗	✗	✗
Choobdari et al.^12^	**✓**	✗	✗	✗	✗	**✓**	**✓**	**✓**	✗	✗	✗
Wang et al.^13^	**✓**	✗	✗	**✓**	✗	✗	**✓**	✗	✗	**✓**	✗
Wang et al.^14^	**✓**	✗	✗	**✓**	✗	✗	**✓**	**✓**	✗	✗	✗
Hu et al.^15^	**✓**	✗	✗	✗	✗	✗	**✓**	✗	✗	✗	✗
Shi et al.^16^	**✓**	✗	✗	✗	✗	**✓**	**✓**	✗	✗	✗	✗
Shahbazbegian et al.^17^	**✓**	✗	✗	✗	✗	✗	**✓**	✗	✗	✗	✗
Amini et al.^18^	**✓**	✗	✗	**✓**	✗	✗	**✓**	✗	✗	**✓**	✗
Lari et al.^19^	**✓**	✗	✗	**✓**	✗	**✓**	**✓**	**✓**	✗	✗	✗
Paktinat et al.^20^	**✓**	**✓**	✗	**✓**	✗	**✓**	**✓**	✗	✗	✗	✗
Behzadi et al.^21^	**✓**	✗	✗	✗	✗	✗	✗	**✓**	✗	✗	✗
Wang et al.^22^	**✓**	✗	✗	**✓**	✗	**✓**	✗	✗	✗	✗	✗
Du and Du^23^	**✓**	✗	**✓**	**✓**	✗	✗	✗	✗	✗	✗	✗
Liu et al.^24^	**✓**	✗	✗	**✓**	✗	**✓**	✗	✗	✗	**✓**	✗
**Proposed**	**✓**	**✓**	**✓**	**✓**	**✓**	**✓**	**✓**	**✓**	**✓**	**✓**	**✓**

### Research gaps and main contributions

The comparative review presented in Table 1 identifies the following research gaps.

• Previous studies have examined multi-energy systems, energy hubs, and heterogeneous outage events. However, relatively few studies have considered these aspects together. Among the reviewed works, the study that addresses both multi-energy operation and heterogeneous outages does not also include operating uncertainty, network reconfiguration, electrical and thermal demand response, recovery metrics, or OOS validation. Therefore, the resilience of integrated electricity, gas, and heat distribution systems under different component outage conditions has not yet been examined comprehensively.
• Operating uncertainty has been considered in several studies, but budgeted polyhedral adverse point scheduling has been applied in only one of the reviewed works. That study does not combine the uncertainty model with a multi-energy system, heterogeneous outages, network reconfiguration, thermal demand response, recovery assessment, or OOS validation. As a result, the coordinated scheduling of prescribed component outages under uncertainty in electrical demand, thermal demand, wind generation, and photovoltaic generation remains insufficiently investigated.
• Network reconfiguration, electrical energy storage systems, and electrical demand response have been studied either separately or in limited combinations. However, thermal demand response is not considered in the reviewed studies, and the four flexibility mechanisms have not been evaluated within the same modeling framework. Their individual effects on interruption severity, interruption duration, resilience recovery, and operating cost therefore cannot be compared under identical network, operating, and disturbance conditions.
• Recovery metrics have been considered in some previous studies; however, OOS validation has not been reported in the reviewed literature.

To address these gaps, this paper makes the following contributions.

• A coordinated operational model is developed for an integrated electricity, gas, and heat distribution system containing multiple energy hubs and prescribed heterogeneous outages. The outage model includes the upstream electricity supply, controllable generation units, wind generation units, and electrical distribution branches within a unified mixed-integer linear scheduling framework.
• A budgeted adverse point scheduling procedure is applied to uncertainty in electrical demand, thermal demand, wind generation shortfall, and photovoltaic generation shortfall. The prescribed outage trajectories are modeled separately from the operating uncertainties. An hourly polyhedral support problem and a single dual guided update are then used to determine the adverse uncertainty coordinates included in the scheduling model.
• A controlled case-based assessment is performed for radial network reconfiguration, electrical energy storage, and coordinated electrical and thermal demand shifting. These flexibility mechanisms are examined in separate cases using the same network data, resource configuration, prescribed disturbance conditions, operating constraints, and uncertainty settings. This structure allows the resilience effect of each mechanism to be identified without changing the underlying study conditions.
• The evaluation considers operating cost and Expected Energy Not Supplied (EENS), together with hourly, energy-based, average, nadir, interruption duration, recovery, and bus-level resilience measures. The selected schedules are also evaluated using fixed plan continuous recourse under operating samples located both inside and outside the modeled uncertainty set. Post-solution power loss calculations and numerical feasibility checks are additionally performed.

Accordingly, the contribution of this study is the coordinated modeling, consistent comparison, and validation of these elements within a single operational framework. The paper does not claim to introduce a new general robust optimization theory; instead, it applies the selected methods to provide a structured assessment of multi-energy system resilience under prescribed outages and operating uncertainty.

### Motivation

Power outages can disrupt essential services, industrial activities, transportation systems, and other infrastructure that depends on a continuous electricity supply, resulting in economic losses, reduced productivity, and uneven restoration outcomes across affected communities.^25^ These consequences have increased the importance of distribution system resilience, which reflects the ability of the network to maintain service during a disturbance and recover its performance within an acceptable period.^26^ The growing integration of distributed energy resources provides additional operational flexibility, but it also increases the complexity of system scheduling under uncertain demand and renewable generation.^27^^,^^28^ In particular, the variability of wind and photovoltaic generation may reduce the effectiveness of operating schedules developed from fixed forecasts. Therefore, uncertainty management should be coordinated with network reconfiguration, energy storage, local generation, and demand response to limit service interruptions and support system recovery under adverse operating conditions. This need becomes more important in integrated electricity, gas, and heat distribution systems, where electrical service depends not only on network connectivity but also on energy conversion, storage availability, and cross-carrier coordination. A consistent assessment of these flexibility mechanisms under the same outage and uncertainty conditions is therefore required to identify their distinct effects on interruption severity, duration, recovery, and operating cost.

### Paper organization

The manuscript is organized into results and discussion, followed by limitations of the study, resource availability, author contributions, declarations, figure titles and legends, tables, STAR Methods, and references. The STAR Methods provide the mathematical formulation, test-system data, prescribed disturbances, operating-uncertainty construction, and computational settings.

## Results

This section examines the operating and resilience consequences of the five final cases and the supplementary analyses. C1 is retained as a no-event operating reference. C2 represents the combined-event baseline, whereas C3, C4, and C5 introduce network reconfiguration, ESS, and DR, respectively. Because C1 does not experience the combined disturbance, its cost and loss values provide operating context but should not be interpreted as an equivalent resilience alternative to C2–C5.

The results are ordered from system-level outcomes to hourly behavior, operating-cost structure, energy conversion and network losses, event-specific performance, flexibility operation, sensitivity tests, and numerical validation. Quantities that are visually informative are assigned to figures, while discrete schedules, critical-bus results, small sensitivity changes, and validation checks are retained in tables. The same numerical result is not repeated in both a figure and a table.

The uncertainty results refer to the scheduled solution under the selected budgeted adverse-point construction. They should not be described as an exact min-max robust counterpart because the formal robust counterpart and iterative or exact column-and-constraint generation flags are inactive. Similarly, the reported event averages are descriptive equal-weight summaries rather than probability-weighted expected values.

### Main system-level outcomes

**Figure 1:**
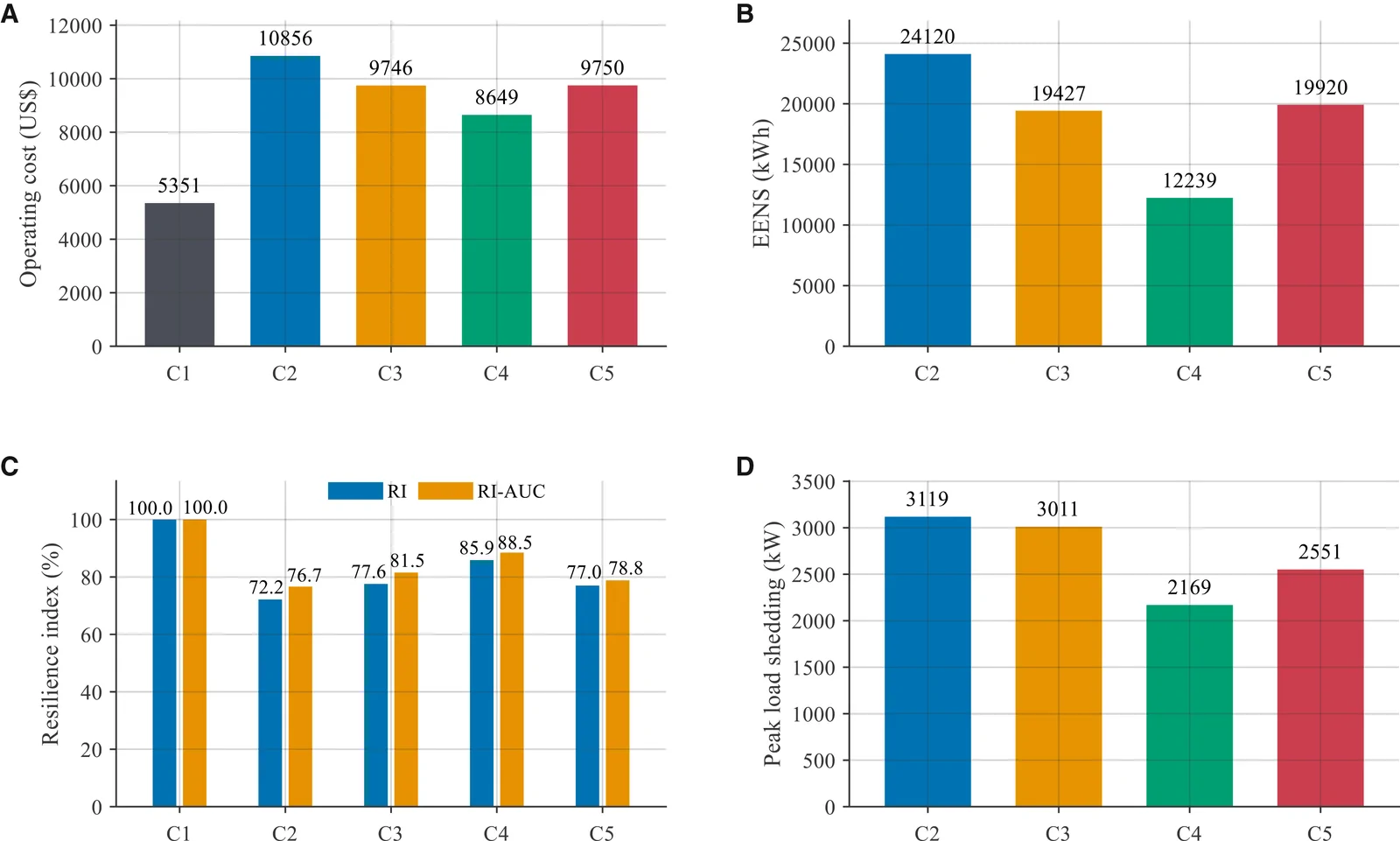
Main system-level outcomes (A) operating cost, (B) EENS, (C) RI and RI-AUC, and (D) peak load shedding.

Figure 1 first compares the principal outcomes. C1 has the lowest operating cost, 5351.17 US$, and no unserved energy because it is the no-event reference. The combined disturbance in C2 raises the operating cost to 10,856.04 US$ and produces 24,119.60 kWh of EENS. The corresponding RI is 72.16%, the RI-AUC is 76.65%, and the peak curtailed load reaches 3118.55 kW. These values define the reference against which the three flexibility mechanisms are evaluated.

Reconfiguration in C3 reduces operating cost by 10.23% and EENS by 19.45% relative to C2. RI increases by 5.42 percentage points, although peak shedding decreases by only 3.44%. This combination indicates that reconfiguration is more effective in changing the duration and location of service interruption than in eliminating the largest instantaneous power deficit. The ESS case C4 provides the lowest cost and EENS among the event-based cases. Its operating cost is 8649.22 US$, 20.33% below C2, while EENS falls by 49.26% and RI rises by 13.71 percentage points. Peak shedding is also 30.44% lower than in C2.

C5 produces an operating cost of 9750.00 US$, an EENS of 19,920.44 kWh, and an RI of 77.01%. Relative to C2, these changes correspond to reductions of 10.19% in cost, 17.41% in EENS, and 18.20% in peak shedding. DR therefore moderates the most stressed hours, but its effect remains below that of ESS because it redistributes demand rather than supplying additional energy. Figure 1 also shows that the ranking based on RI and RI-AUC is consistent: C4 performs best, followed by C3 and C5, while C2 remains the least resilient event-based case.

Table 2 presents the interruption and recovery metrics for C2-C5. The RI nadir occurs at hour 19 in all four cases. C3 reduces the number of shedding hours from 19 to 9, shortens the longest uninterrupted interval from 19 to 5 h, and restores full service 4 h after the nadir. C4 retains 15 shedding hours, but raises the nadir RI to 56.03% and reduces peak shedding to 2169.40 kW. Thus, C3 provides the shortest interruption duration, whereas C4 provides the largest reduction in interruption severity.

**Table 2:** Interruption and recovery metrics for the event-based cases

Case	Nadir RI (%)	Peak shedding (kW)	Shedding hours	Longest interval (h)	Time to full service after nadir (h)	End-horizon RI (%)
C2	36.788	3118.55	19	19	5	100
C3	38.965	3011.19	9	5	4	100
C4	56.027	2169.40	15	15	5	100
C5	40.132	2550.84	19	19	5	100

### Hourly operational response

**Figure 2:**
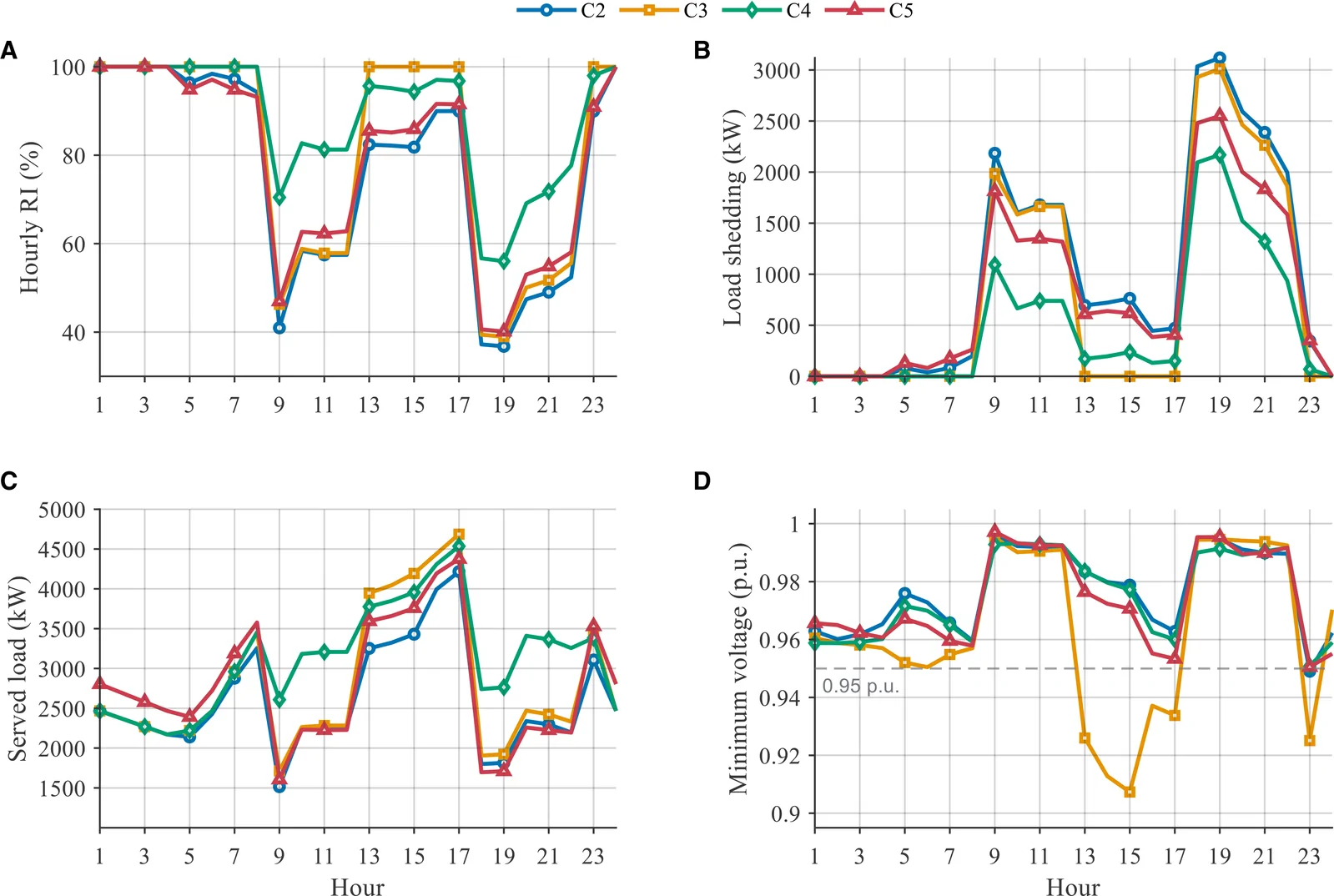
Hourly operational response of C2–C5 (A) RI, (B) load shedding, (C) served load, and (D) minimum bus voltage.

Figure 2 resolves the aggregate indices into hourly trajectories. Two stressed periods are visible. The first is concentrated around hours 9–12, and the second begins at hour 18 and reaches its maximum at hour 19. C2 shows the deepest reduction in hourly RI during both periods. At hour 19, only 1814.95 kW of 4933.50 kW demand is served, which explains the 3118.55 kW peak deficit. The system then recovers progressively and restores full service at the end of the horizon.

The C3 trajectory confirms the duration-oriented role of reconfiguration. Load shedding is eliminated during hours 13–17 and again at hours 23–24, which separates the disturbance into shorter intervals. However, the remaining deficits during hours 18–22 are still large; this is why the EENS reduction is smaller than the reduction in interruption duration. C4 maintains the highest RI throughout most stressed hours. During hours 9–12, shedding remains between 665.39 and 1092.88 kW, compared with 1601.50–2184.38 kW in C2. During the second stressed period, ESS discharge reduces the deficit at hour 19 to 2169.40 kW.

The C5 profile lies between C2 and C4. Demand shifting reduces the second-period peak and raises served load, but the 19 shedding hours remain because the shifted energy must be restored in other periods. The voltage panel also separates the network effects of the mechanisms. C2, C4, and C5 remain close to 0.95 p.u., with minimum values of 0.949044, 0.950754, and 0.950543 p.u., respectively. C3 reaches 0.907342 p.u. under its original voltage setting. This low value does not indicate an infeasible solved case under the original formulation, but it explains why C3 is the only case that responds materially when the explicit 0.95-p.u. sensitivity is imposed later.

### Operating-cost structure

**Figure 3:**
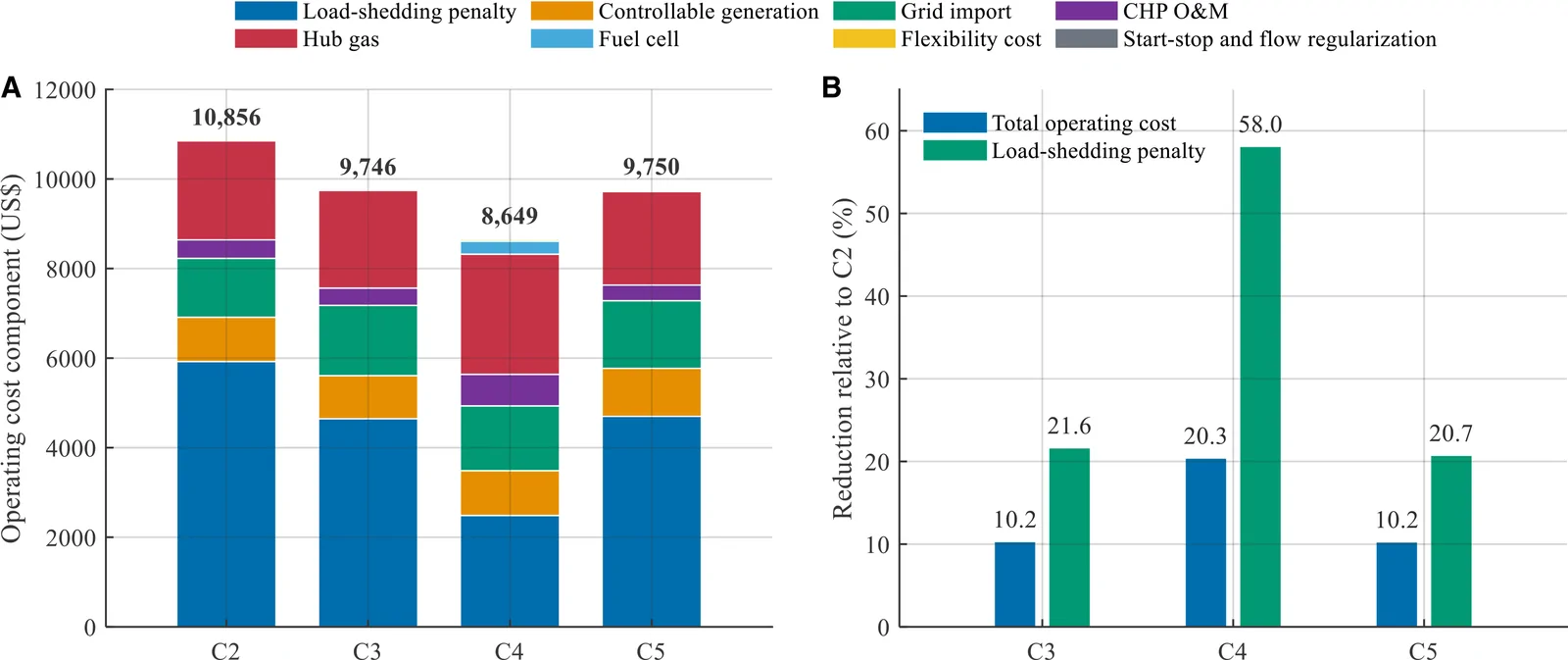
Operating-cost structure (A) percentage contribution of each cost component and (B) reductions in total cost and load-shedding penalty relative to C2.

Figure 3 identifies the components responsible for the economic differences. In C2, the load-shedding penalty is 5923.96 US$, accounting for 54.57% of total operating cost. This confirms that the economic ranking is driven primarily by the ability to preserve service rather than by uniform reductions in generation or fuel expenditures. C3 lowers the penalty to 4646.10 US$, and C5 lowers it to 4700.25 US$. Their total-cost reductions are therefore similar even though the mechanisms are different.

C4 reduces the shedding penalty to 2486.52 US$, only 28.75% of total cost. This reduction more than offsets higher CHP, fuel-cell, gas, and storage-cycling costs. The case uses 287.81 US$ of fuel-cell operating and fuel costs and 39.27 US$ of storage degradation costs, while hub-gas cost increases to 2682.89 US$. The result should therefore not be interpreted as a uniform decrease in all cost components. It is a redistribution toward controllable energy supply that avoids a larger interruption penalty.

The right image of Figure 3 separates total-cost reduction from the reduction in shedding penalty. C4 reduces the shedding penalty by approximately 58.03% relative to C2, compared with 21.57% in C3 and 20.65% in C5. These values explain why C4 achieves a 20.33% reduction in total operating cost, nearly twice the reduction obtained in C3 and C5. All reported costs are operating costs; capital expenditure, installation cost, and life cycle replacement cost are outside the scope of this comparison.

### Aggregate energy and network operation

**Figure 4:**
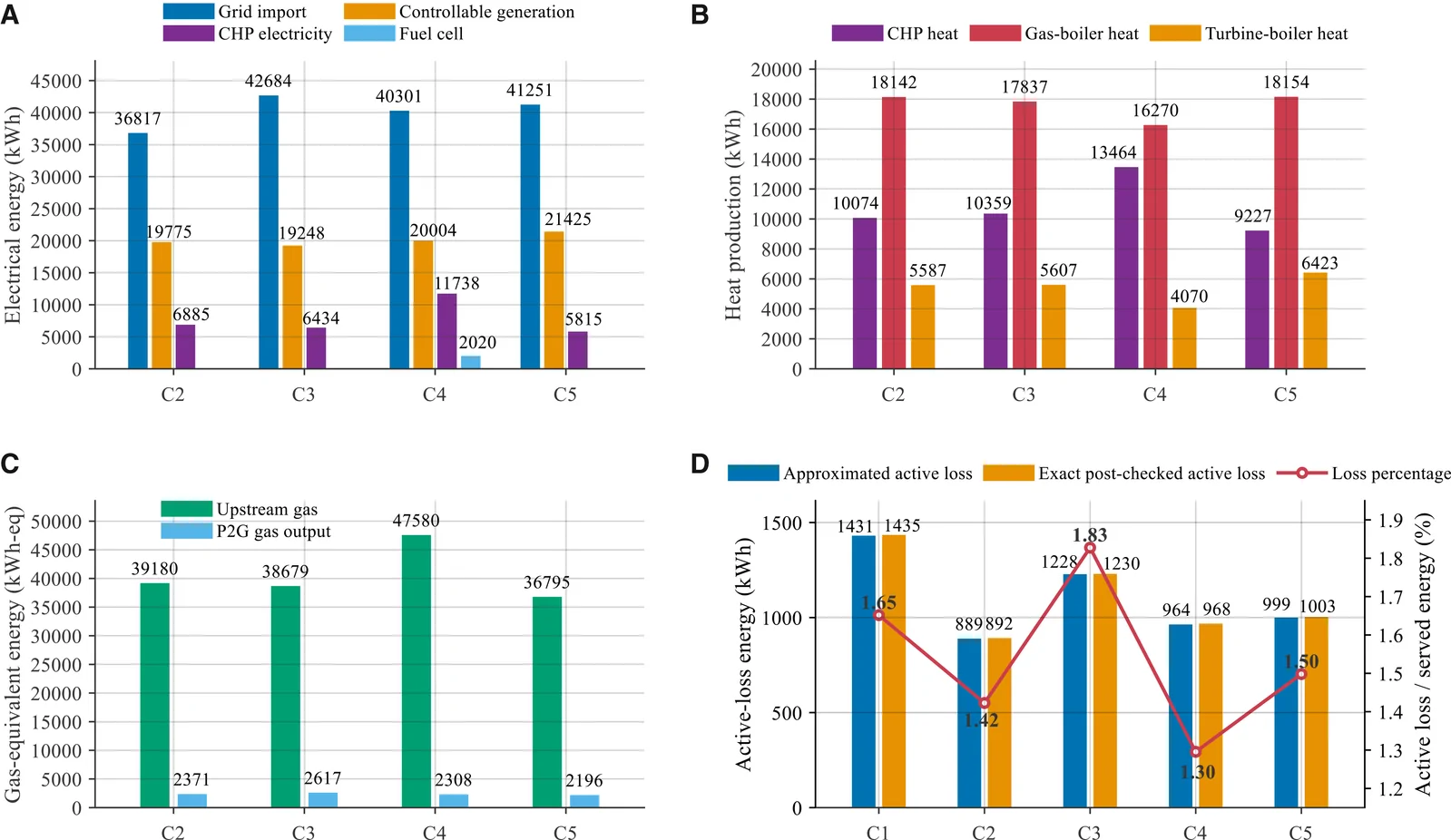
Aggregate energy and network operation (A) electricity supply, (B) heat production, (C) upstream-gas and P2G contributions, and (D) approximated and exact active-loss energy with the active-loss percentage.

Figure 4 examines how each event-based case supplies electricity and heat. C3 imports 42,684.15 kWh from the upstream grid, 15.94% more than C2, while controllable generation and CHP electricity are slightly lower. This pattern is consistent with reconfiguration: the mechanism changes feasible delivery paths and reconnects load, but it does not add an energy buffer. C4 combines 40,301.04 kWh of grid imports with 20,004.20 kWh of controllable generation, 11,738.30 kWh of CHP electricity, and 2019.80 kWh of fuel-cell generation. The larger local conversion contribution supports the substantial reduction in EENS.

The thermal results show the same cross-carrier adjustment. C4 raises CHP heat to 13,463.72 kWh and reduces gas-boiler and turbine-boiler heat to 16,269.60 and 4069.67 kWh. This dispatch requires 47,579.60 kWh-eq of upstream gas, 21.44% above C2. The higher gas use should therefore be interpreted together with the reduction in unserved electricity; it is a substitution from interruption toward gas-supported CHP and fuel-cell operation. C5 has the lowest upstream gas purchase among the event-based cases, 36,795.44 kWh-eq, but relies more heavily on controllable electrical generation and demand shifting.

The active-loss results, included explicitly in Figure 4D, are also important. Approximate active-loss energy is 889.32 kWh in C2, 1228.18 kWh in C3, 963.74 kWh in C4, and 999.43 kWh in C5. C3 has the highest absolute and relative losses among the event cases because reconfiguration serves more energy through altered and, at some hours, longer electrical paths. Its active losses equal 1.83% of served energy. C4 has a higher absolute loss than C2 but the lowest loss ratio, 1.30%, because it serves substantially more load. This distinction prevents a misleading conclusion based only on absolute loss energy.

The exact post-checked active losses remain close to the approximated values: 891.82 kWh in C2, 1229.83 kWh in C3, 967.70 kWh in C4, and 1003.35 kWh in C5. The no-event reference C1 has 1430.83 kWh of approximate active loss and 1435.09 kWh after the exact check because it serves the full daily demand and imports from the grid during all 24 h. C1 should therefore not be used to infer that the disturbed cases are intrinsically more efficient; their lower absolute losses partly reflect unserved energy and fewer grid-import hours.

Table 3 lists the three buses with the highest EENS in each event-based case. The location of the largest service deficit changes with the flexibility mechanism: i20 is most affected in C2 and C5, whereas i25 is most affected in C3. In C4, the maximum bus-level EENS decreases to 1292.33 kWh at i7, compared with 1940.40 kWh at i20 in C2 and 2304.60 kWh at i25 in C3. The buses with the largest EENS do not consistently coincide with those having the lowest voltage, indicating that service criticality and voltage criticality represent different aspects of network performance.

**Table 3:** Three buses with the largest EENS in each event-based case

Case	Bus	Bus EENS (kWh)	Bus RI (%)	Minimum voltage (p.u.)
C2	i20	1940.40	44.191	0.992721
C2	i25	1755.14	78.366	0.975756
C2	i7	1740.20	54.954	0.965644
C3	i25	2304.60	71.593	0.974006
C3	i7	1740.20	54.954	0.963399
C3	i8	1740.20	54.954	0.957262
C4	i7	1292.33	66.548	0.967450
C4	i29	1178.19	49.170	0.957198
C4	i19	1044.45	39.920	0.997506
C5	i20	1730.88	50.217	0.991927
C5	i7	1502.90	61.097	0.965479
C5	i8	1502.90	61.097	0.963289

### Event-specific comparison and ESS effectiveness

**Figure 5:**
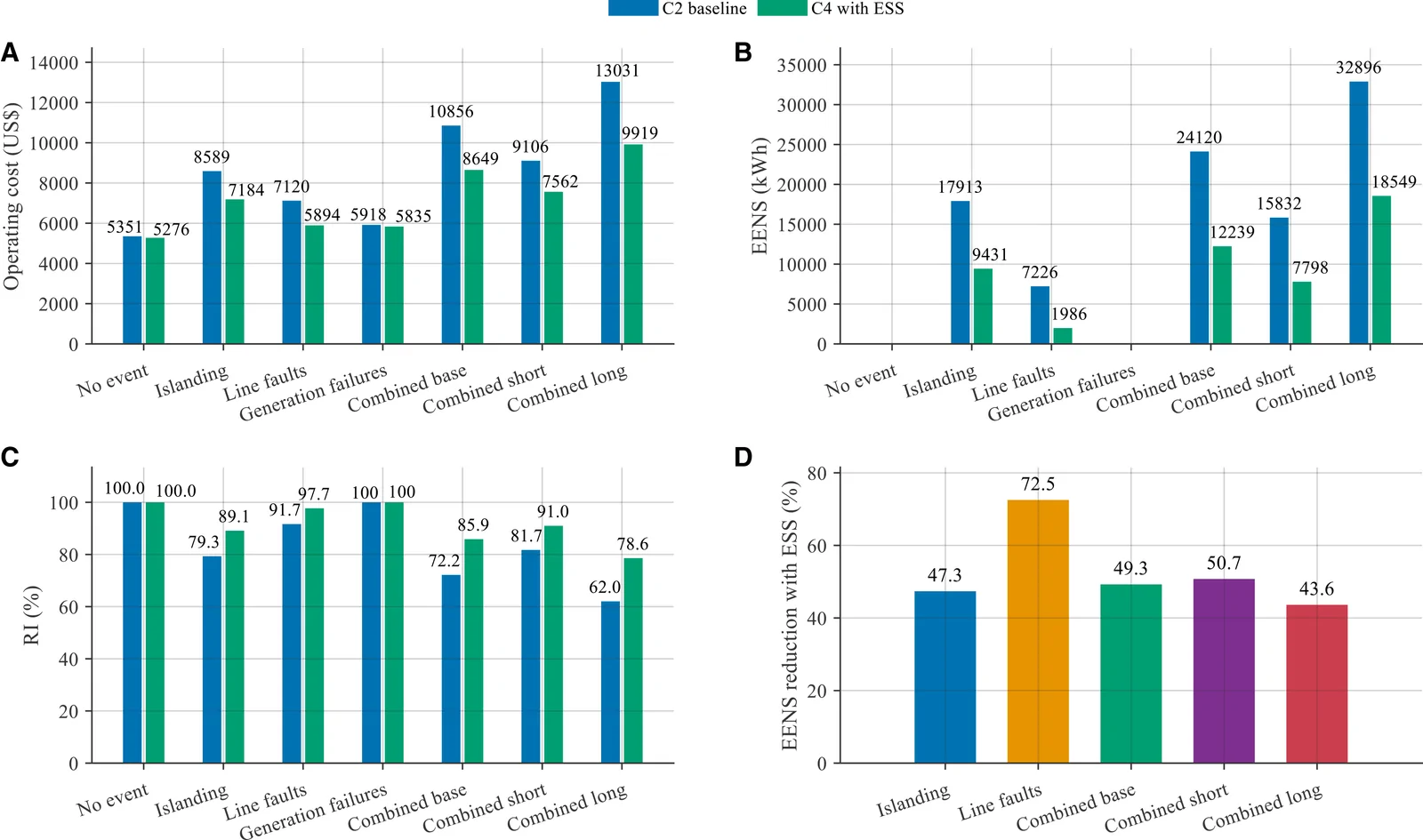
Event-specific comparison of C2 and C4 (A) operating cost, (B) EENS, (C) RI, and (D) EENS reduction obtained with ESS.

Figure 5 compares C2 and C4 under seven event configurations. The no-event and generation-failure-only cases produce zero EENS in both models. This result means that the upstream grid and remaining local resources can compensate for the unavailable generator and wind unit when network access is retained. Islanding is more severe than the line-fault event because it removes upstream support, whereas line faults preserve some supply paths and can be managed through the remaining network.

The combined events produce the largest deficits. In C2, EENS increases from 15,832.46 kWh in the short-combined event to 24,119.60 kWh in the base event and 32,895.67 kWh in the long event. The corresponding RI values fall from 81.72% to 72.16% and 62.03%. This ordering is internally consistent with event duration. In C4, the same ordering remains, but EENS is reduced to 7798.34, 12,239.04, and 18,548.86 kWh, respectively.

ESS reduces EENS by 47.35% under islanding, 72.51% under line faults, 49.26% in the combined-base event, 50.74% in the short-combined event, and 43.61% in the long-combined event. The lower percentage improvement in the long event is plausible because the finite stored-energy inventory must be distributed over a longer period. The event study therefore supports the C4 result without implying that storage removes all duration effects.

Table 4 presents the corresponding recovery and loss-approximation results. Islanding, line faults, and the short-combined event return to full service 1 h after the disturbance ends in both C2 and C4. The base and long combined events continue to the end of the scheduling horizon, so a separate post-event recovery time is not defined. C4 nevertheless ends the long event at full service, whereas C2 finishes with an RI of 89.97%. Across all event configurations, the loss-approximation error remains below 0.61%.

**Table 4:** Event-specific recovery and loss-approximation results

Event	Case	Nadir RI (%)	End-horizon RI (%)	Recovery after event (h)	Full service by horizon	Loss error (%)
No event	C2	100.000	100.000	–	yes	0.297
No event	C4	100.000	100.000	–	yes	0.461
Islanding	C2	49.850	100.000	1	yes	0.133
Islanding	C4	69.029	100.000	1	yes	0.600
Line faults	C2	81.793	100.000	1	yes	0.001
Line faults	C4	94.329	100.000	1	yes	0.036
Generation failures	C2	100.000	100.000	–	yes	0.178
Generation failures	C4	100.000	100.000	–	yes	0.609
Combined base	C2	36.788	100.000	–	yes	0.280
Combined base	C4	56.027	100.000	–	yes	0.410
Combined short	C2	37.225	100.000	1	yes	0.455
Combined short	C4	56.764	100.000	1	yes	0.498
Combined long	C2	36.788	89.967	–	no	0.415
Combined long	C4	56.051	100.000	–	yes	0.404

### Sensitivity to the budgeted adverse-point level

**Figure 6:**
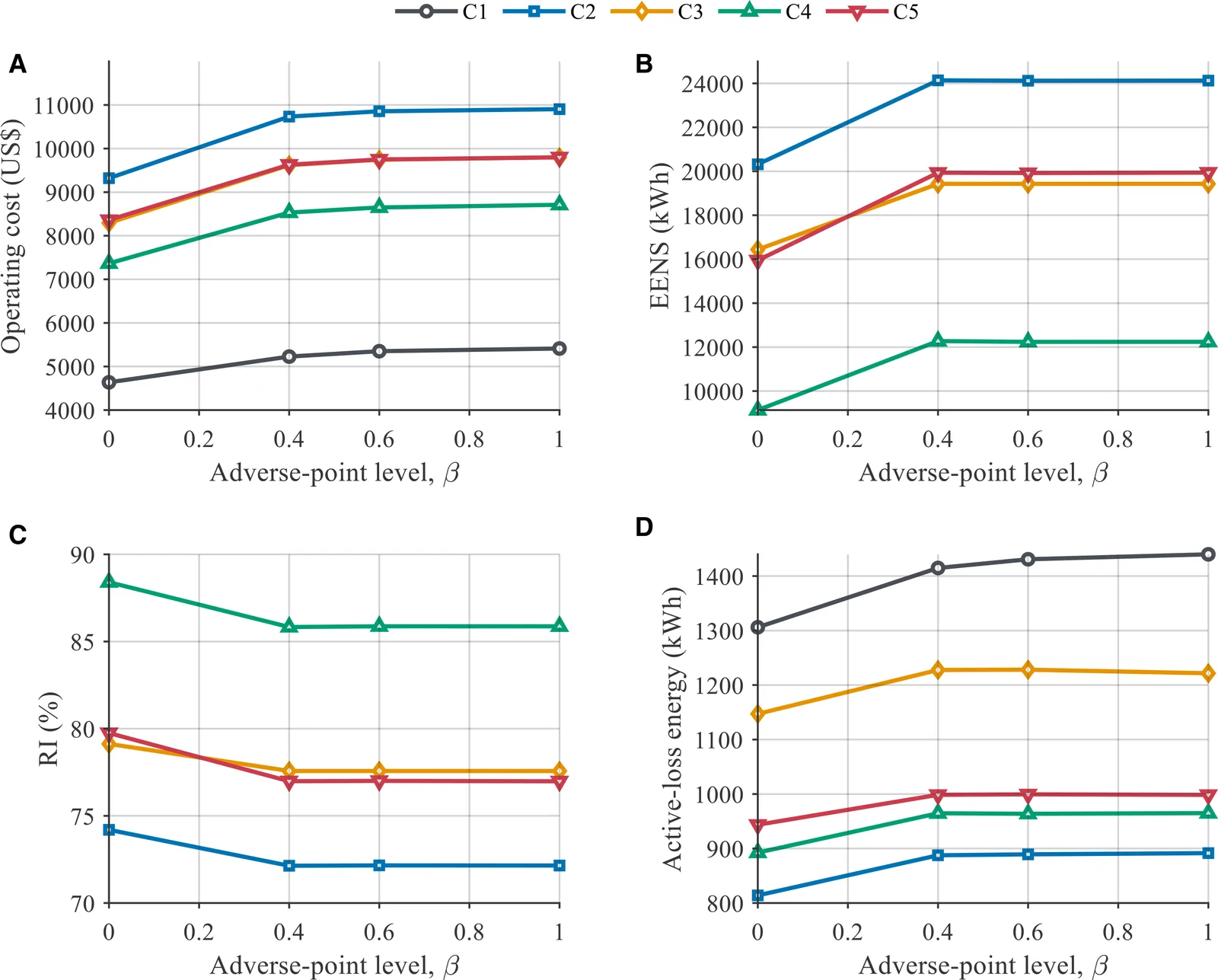
Sensitivity to the budgeted adverse-point level (A) operating cost, (B) EENS, (C) RI, and (D) active-loss energy.

Figure 6 examines four adverse-point levels, *β* = 0, 0.4, 0.6, and 1.0. The largest change generally occurs between *β* = 0 and *β* = 0.4. For C2, operating cost increases from 9320.38 to 10,733.74 US$ and EENS from 20,318.92 to 24,136.95 kWh. Beyond *β* = 0.4, the response is comparatively flat: the C2 cost changes by only 1.59% from *β* = 0.4 to 1.0, while EENS remains close to 24,120 kWh. Similar plateaus occur in C3–C5.

C4 remains the best event-based case over the full range. Its EENS is 9134.65 kWh at *β* = 0 and approximately 12,240 kWh at *β* = 1.0, while RI remains above 85.8% for β ≥ 0.4. The ordering C4, C3/C5, and C2 is therefore not an artifact of the selected *β* = 0.6 point. The sensitivity should still be interpreted as a sequence of budgeted adverse-point schedules, not as a probability distribution or a formal robust frontier.

The active-loss panel, which was omitted in earlier drafts, provides an additional check on network behavior. At *β* = 0.6, it reproduces the main-case loss values exactly. Increasing β changes active-loss energy only modestly after *β* = 0.4 in C2, C4, and C5. C3 shows a small non-monotonic response between *β* = 0.6 and 1.0, reflecting topology and dispatch changes rather than a direct proportional relation between uncertainty level and network loss.

### Operation of the flexibility mechanisms

#### C3 reconfiguration operation

**Figure 7:**
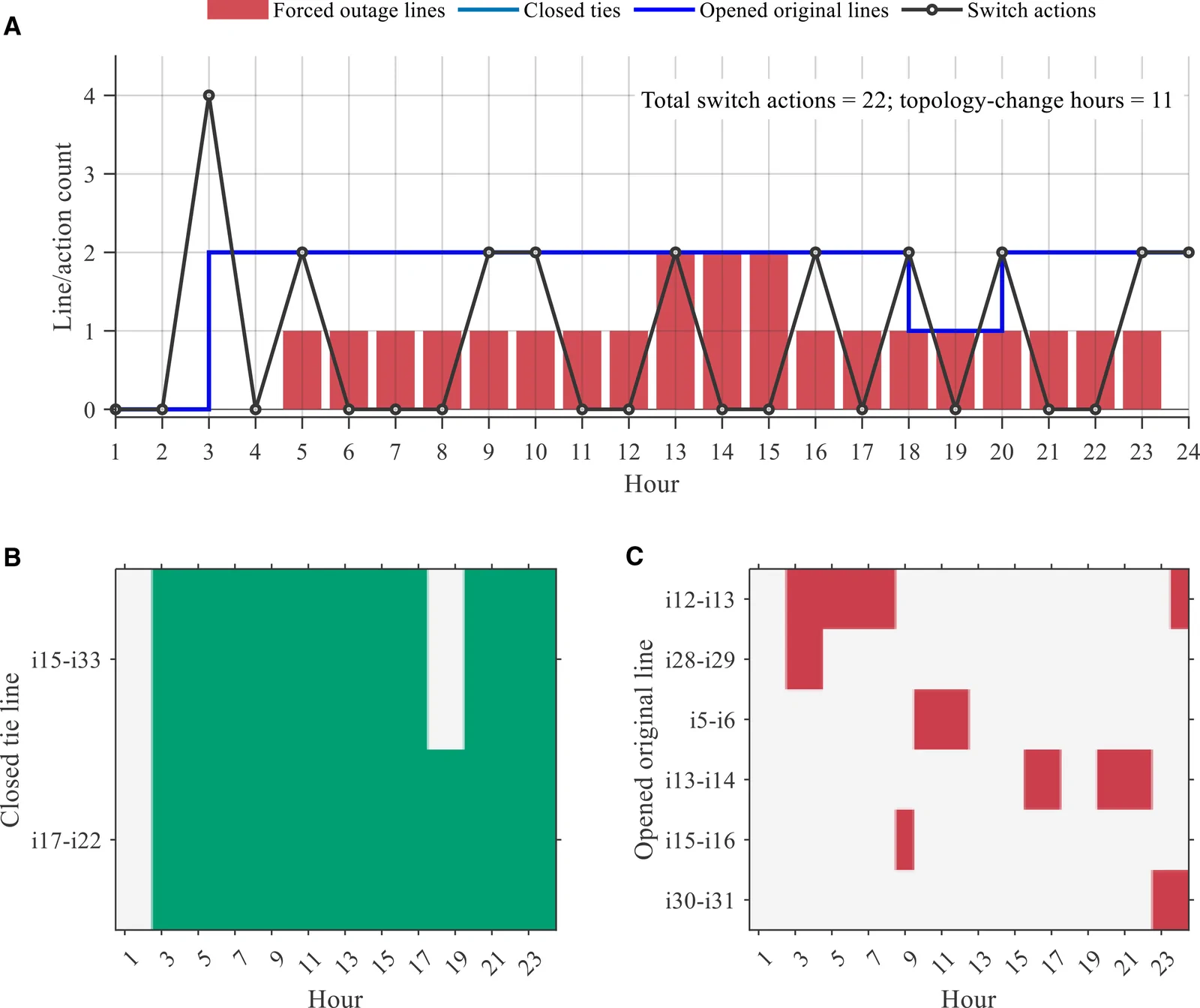
C3 reconfiguration operation (A) hourly outage, tie, open-line, and switching counts, (B) closed tie-line status, and (C) opened original-line status.

Figure 7 shows the reconfiguration schedule. The two tie lines 15–33 and 17–22 are closed after hour 2 to preserve radial supply paths around forced outages. The first tie remains closed for 20 h and the second for 22 h. A total of 22 switching actions is recorded, and topology-change flags occur in 11 h. The model normally opens two original lines while both ties are active, but this number temporarily falls to one during hours 18–19 when only one tie remains closed.

The heatmaps identify the specific original lines used to maintain radiality. Line 12–13 is opened for 7 h, 13–14 for 5 h, 5–6 for 3 h, and 28–29 and 30–31 for 2 h each. The schedule explains why C3 removes shedding over several intervals and reduces the longest interruption to 5 h. It also explains the higher active-loss ratio: power is redirected through alternative paths whose primary purpose is service restoration rather than minimum-loss operation.

#### C4 energy-storage operation

**Figure 8:**
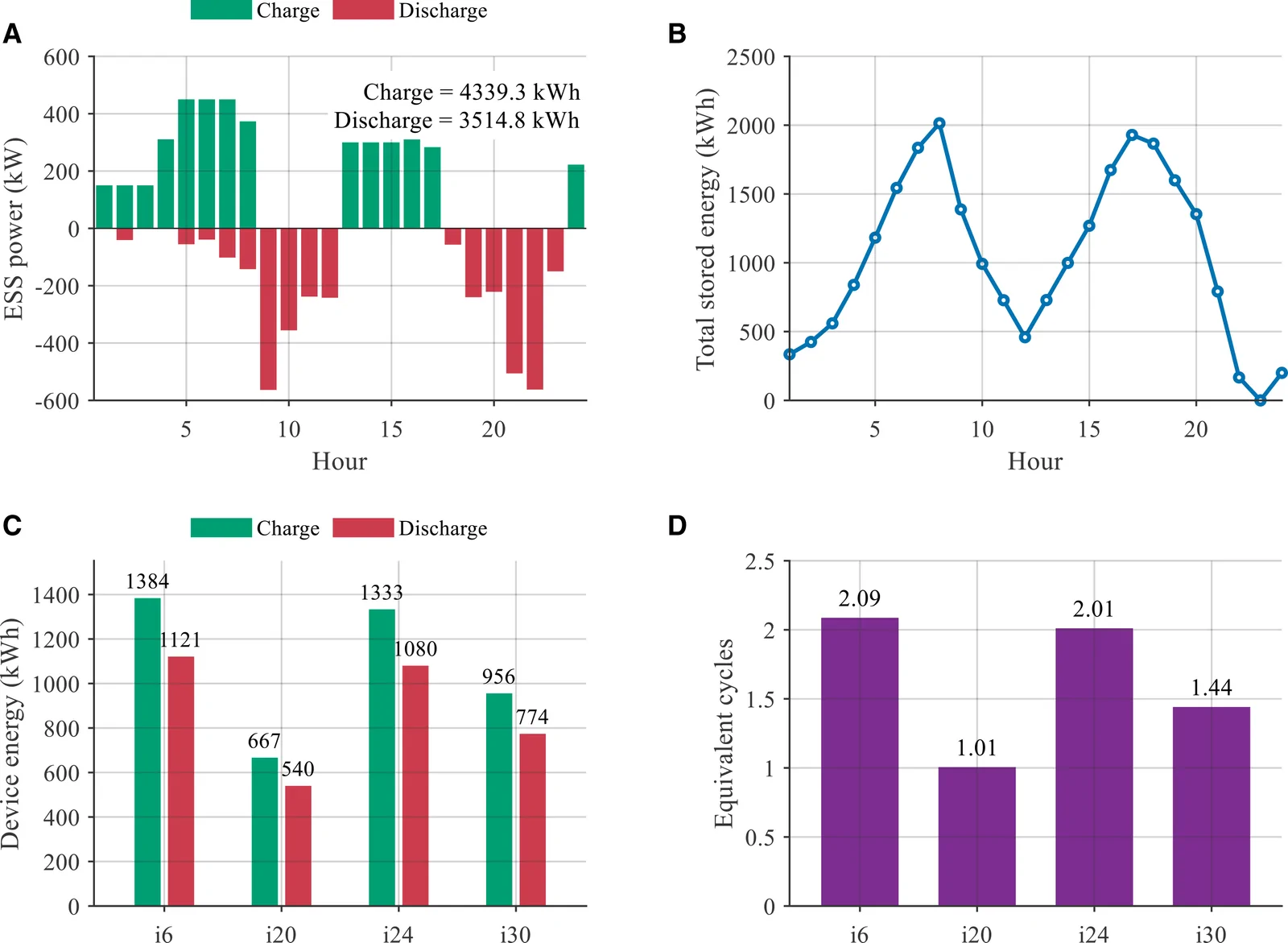
C4 energy-storage operation (A) hourly charge and discharge, (B) aggregate stored energy, (C) device-level charged and discharged energy, and (D) equivalent cycles.

Figure 8 relates storage operation to the two stressed periods. The four ESS units charge by 4339.26 kWh and discharge by 3514.80 kWh over the horizon. Charging is concentrated before the first deficit, during hours 1–8, and again during hours 13–17. Discharge then supports hours 9–12 and 18–23. The aggregate discharge peaks at 563.48 kW in hour 9 and 562.31 kW in hour 22. This timing is consistent with the lower hourly shedding in Figure 2.

The units at i6 and i24 provide the largest throughputs, 2504.50 and 2413.33 kWh, with 2.09 and 2.01 equivalent cycles. The i20 and i30 devices provide smaller contributions. All four units return to 50 kWh at the end of the horizon, and the terminal residual is zero. The absence of simultaneous charging and discharging at the asset-hour level confirms that the aggregate profile is created by physically consistent individual schedules.

#### C5 demand-response operation

**Figure 9:**
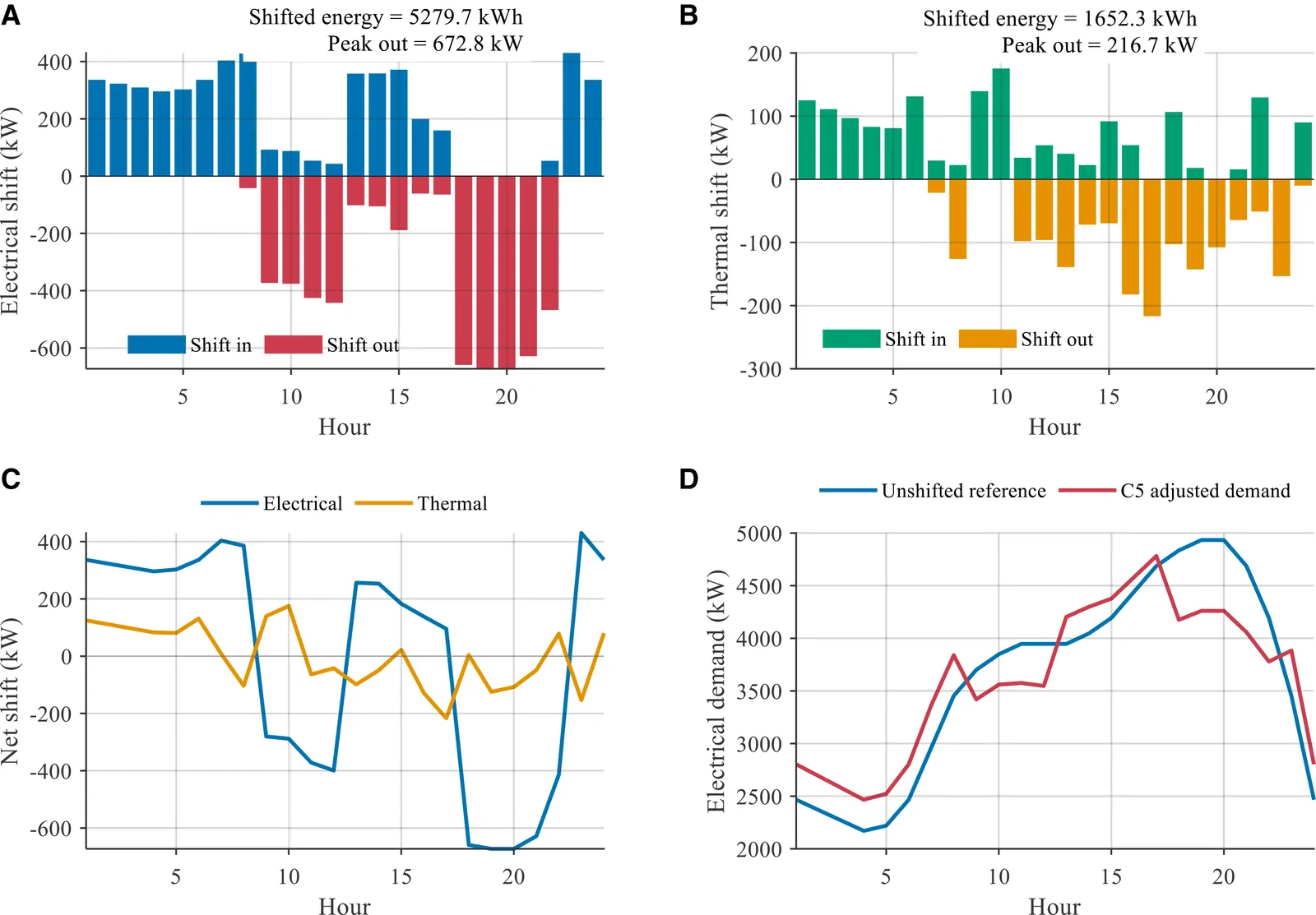
C5 demand-response operation (A) electrical shift-in and shift-out, (B) thermal shift-in and shift-out, (C) net electrical and thermal shifts, and (D) unshifted and adjusted electrical-demand profiles.

Figure 9 shows that electrical demand is moved into the early and intermediate hours and away from the main deficit periods. The electrical program shifts 5279.70 kWh in each direction, with a maximum shift-out of 672.75 kW during hours 19 and 20. The adjusted demand is therefore lower than the unshifted reference during the evening stress period, which explains the 18.20% reduction in peak shedding relative to C2.

Thermal DR is smaller, with 1652.27 kWh shifted in each direction. The maximum thermal shift-out is 216.70 kW at hour 17. Electrical and thermal neutrality residuals are below 10^−11^ kWh, and no bus-hour or hub-hour simultaneous shift-in/shift-out condition is reported. The response therefore represents energy-neutral relocation rather than permanent demand reduction. Because the shifted energy must be recovered, C5 retains 19 shedding hours and does not match the EENS reduction achieved by ESS.

Table 5 summarizes the device- and network-level operating details of C3-C5. The C3 entries quantify tie-line use, opened-line durations, and switching actions. The C4 entries confirm the discharge duration, terminal energy, and device power levels of the four ESS units. The C5 entries report the maximum electrical and thermal shifts together with the neutrality and non-overlap checks. These results link the aggregate performance gains to the schedules of the individual flexibility resources.

**Table 5:** Mechanism-specific operating details not repeated in the figures

Mechanism	Asset/item	Metric	Value	Unit
C3 reconfiguration	i15–i33	tie closed	20	h
C3 reconfiguration	i17–i22	tie closed	22	h
C3 reconfiguration	All switches	total switching actions	22	actions
C3 reconfiguration	Topology	topology-change flags	11	hours
C3 reconfiguration	i12–i13	original line opened	7	h
C3 reconfiguration	i13–i14	original line opened	5	h
C4 ESS	i6	discharge duration; final energy	9; 50	h; kWh
C4 ESS	i20	discharge duration; final energy	6; 50	h; kWh
C4 ESS	i24	discharge duration; final energy	9; 50	h; kWh
C4 ESS	i30	discharge duration; final energy	8; 50	h; kWh
C4 ESS	all units	terminal energy residual	0	kWh
C4 ESS	each unit	peak charge/discharge power	150/150	kW
C5 DR	electrical	peak shift-in/shift-out	430.02/672.75	kW
C5 DR	thermal	peak shift-in/shift-out	175.43/216.70	kW
C5 DR	electrical	neutrality residual	9.09 × 10^−12^	kWh
C5 DR	thermal	neutrality residual	6.82 × 10^−13^	kWh
C5 DR	both carriers	simultaneous in/out count	0	asset-hours

### Voltage-limit and flexibility-cost sensitivity

Table 6 summarizes the voltage-limit and flexibility-cost sensitivities. Imposing a 0.95-p.u. minimum voltage has negligible effects on C2, C4, and C5: their cost changes are approximately 0.10, 0.001, and 0.010 US$, respectively, and their resilience indices remain unchanged at the reported precision. These cases already operate at or close to the tested voltage limit.

**Table 6:** Voltage-limit and flexibility-cost sensitivity results

Test	Case	Cost (US$)	EENS (kWh)	RI (%)	Active loss (kWh)	Minimum voltage (p.u.)	Flexibility activity
Vmin = 0.95	C2	10856.14	24119.63	72.1586	888.95	0.950000	no flexibility activity
Vmin = 0.95	C3	9808.22	19503.03	77.4876	1021.93	0.950000	10 shedding h; 22 switch actions
Vmin = 0.95	C4	8649.22	12239.04	85.8724	963.76	0.950000	ESS throughput 7854.05 kWh
Vmin = 0.95	C5	9750.01	19920.56	77.0056	999.42	0.950544	Elec./thermal shift 5279.70/1652.24 kWh
ESS cost ×1.5	C4	8668.86	12239.04	85.8724	963.74	0.950754	ESS throughput 7854.05 kWh
ESS cost ×2.0	C4	8688.40	12239.04	85.8724	959.98	0.950754	ESS throughput 7762.89 kWh
DR cost ×1.5	C5	9762.13	19920.78	77.0054	998.75	0.950543	Elec./thermal shift 5243.66/1569.57 kWh
DR cost ×2.0	C5	9774.15	19921.20	77.0049	998.17	0.950543	Elec./thermal shift 5225.71/1557.74 kWh

C3 shows the only material voltage response. Raising its minimum voltage from 0.907342 to 0.95 p.u. increases operating cost by 62.47 US$ (0.64%), raises EENS by 75.66 kWh (0.39%), and lowers RI by 0.087%. Active-loss energy decreases from 1228.18 to 1021.93 kWh because the stricter limit changes both topology and dispatch. This loss reduction is accompanied by a small increase in cost and unserved energy and is therefore not an independent efficiency gain.

The flexibility-cost tests produce only small changes in system performance. Increasing ESS cycling cost by 50% leaves the schedule unchanged and raises total cost by 0.227%. Doubling the cost reduces ESS throughput by 1.16% and increases total cost by 0.453%, with no change in EENS or RI. In C5, 50% and 100% increases in DR cost raise total cost by 0.124% and 0.248%. Electrical shifting decreases by 0.68% and 1.02%, while thermal shifting decreases by 5.00% and 5.72%. Thermal response is more sensitive to its operating cost, but the system-level ranking is unchanged.

### Out-of-sample and numerical validation

Table 7 presents the out-of-sample results and the numerical audit. All 40 samples are solved to optimality for each event-based case, including 20 samples inside and 20 outside the modeled uncertainty budget. The adverse-point solution dominates the in-budget samples in every case. The outside-budget samples are used as additional stress tests rather than as empirical realizations, so the reported statistics support comparative validation and are not probabilistic forecasts.

**Table 7:** Out-of-sample and numerical-validation summary

Case	Optimal OOS solves	Worst OOS cost (US$)	Worst OOS EENS (kWh)	Minimum OOS RI (%)	Loss error (%)	Maximum point error (kW)	Minimum voltage (p.u.)	Voltage violations	Overall audit
C1	–	–	–	–	0.297	0.417	0.960727	0	pass
C2	40/40	10427.90	23053.59	72.5928	0.280	0.543	0.949044	0	pass
C3	40/40	9344.63	18605.89	77.9208	0.135	0.367	0.907342	0	pass
C4	40/40	8303.32	11342.76	86.5489	0.410	0.591	0.950754	0	pass
C5	40/40	9364.02	18742.71	77.7680	0.391	0.940	0.950543	0	pass

C4 retains the strongest out-of-sample performance, with a worst sampled cost of 8303.32 US$, a worst EENS of 11,342.76 kWh, and a minimum RI of 86.55%. The corresponding worst EENS values are 23,053.59 kWh in C2, 18,605.89 kWh in C3, and 18,742.71 kWh in C5. The out-of-sample results therefore preserve the ordering obtained from the main adverse-point analysis.

The numerical audit confirms the consistency of the reported solutions. All main runs have model and solve status 1. For C2-C5, the loss-approximation error ranges from 0.135% to 0.410%, and the maximum pointwise loss error remains below 1 kW. Active-power, reactive-power, thermal, gas, fuel-cell, and terminal-storage balance residuals are zero at the reported tolerance. No voltage-limit violation, simultaneous grid import and export, simultaneous ESS charging and discharging, DR overlap, uncertainty-budget violation, or self-audit failure is recorded.

The retained plan is case-specific. C2 retains the generator and CHP commitment schedules. C3 additionally retains the hourly radial switching schedule. C4 retains the unit commitments and the ESS charging, discharging, and reactive-support schedules. C5 retains the unit commitments and the electrical and thermal demand-shifting schedules. Generation levels, energy-conversion outputs, upstream exchange, network flows, renewable curtailment, and emergency load shedding remain available for continuous redispatch. Hence, the stress assessment evaluates recourse around the retained plan rather than performing a scenario-specific full reoptimization.

The results separate three forms of flexibility. Reconfiguration primarily changes interruption duration and restores feasible supply paths; it reduces shedding to 9 h and the longest interval to 5 h, but it does not create energy and can increase network losses through alternative routes. DR reduces stressed-hour demand and peak shedding with a small explicit operating cost, but the shifted demand must be recovered, so its EENS improvement is moderate. ESS provides both temporal energy transfer and direct support during the most severe hours, leading to the lowest operating cost, lowest EENS, highest RI, and lowest active-loss percentage among the event-based cases.

The event, uncertainty, voltage, cost, OOS, and numerical analyses support the same ranking without suggesting that one mechanism is superior for every metric. C3 is preferable when shortening interruption duration is the priority. C5 provides a moderate improvement with limited direct cost. C4 gives the strongest reduction in total unserved energy and peak deficit, but it uses more gas-supported conversion, and its results concern operating performance only. A planning conclusion about investment would require capital cost, asset lifetime, replacement, and implementation constraints, which are not included in the present results.

### Computational performance

Table 8 reports the final model dimensions and computational performance obtained directly from the GAMS Solve Summary outputs. The model-status and solve-status codes were equal to 1 for all five cases, corresponding to an optimal model and normal solver completion. Solver time denotes the cumulative CPLEX solution time recorded for the case-specific support, screening, loss-refinement, and final scheduling sequence, whereas elapsed time denotes the associated wall-clock time.

**Table 8:** Model dimensions and computational performance of the five main cases

Case	Case definition	Equations	Variables	Discrete variables	Model/solve status	Final relative MIP gap	Solver time (s)	Elapsed time (s)	Loss-refinement iterations
C1	no-event reference	20,178	15,050	168	optimal/normal completion	EPS	3.701	21.314	8
C2	combined-outage baseline	20,178	15,050	63	optimal/normal completion	EPS	3.969	24.535	8
C3	radial network reconfiguration	25,002	18,698	155	optimal/normal completion	EPS	252.719	276.490	12
C4	electrical energy storage	20,566	15,434	155	optimal/normal completion	2.17 × 10^−9^	5.078	30.426	8
C5	electrical and thermal demand shifting	21,991	16,826	133	optimal/normal completion	EPS	15.171	52.184	13

Notes: EPS is the GAMS representation of a value that is numerically zero at the displayed precision. Model status 1 denotes an optimal model, and solve status 1 denotes normal completion. The reported times and iteration counts are taken directly from the final case-specific Solve Summary outputs.

The computational results confirm successful solution of all five formulations. C3 is the largest and most computationally demanding case, with 25,002 equations, 18,698 variables, and 252.719 s of cumulative solver time. This increase is consistent with the activation of hourly network-topology decisions and the associated connectivity, radiality, and switching constraints. C4 adds the ESS operating variables but remains close to the baseline model in scale and requires 5.078 s of solver time. C5 contains 21,991 equations and 16,826 variables and records the largest number of loss-refinement iterations, 13, while its cumulative solver time remains 15.171 s. C1 and C2 have the same total numbers of equations and variables, although their reported discrete-variable counts differ because their final case implementations activate and fix different case-specific decisions. The discrete-variable count should therefore not be interpreted as the sole indicator of computational difficulty. All cases satisfy the prescribed relative optimality requirement and complete well below the 1800 s time limit.

## Discussion

This paper examined the resilient operation of an electricity, gas, and heat distribution system in which energy hubs coordinate local generation, conversion, and flexibility resources under prescribed heterogeneous outages and operating uncertainty. The results distinguish three complementary resilience functions. Network reconfiguration primarily restores feasible delivery paths and limits interruption duration. Energy storage systems provide temporal energy transfer and direct support during supply-deficient hours. Electrical and thermal demand response reshape the demand profile without introducing additional energy. This distinction is important because no single system-level index fully captures the operational value of these mechanisms. Under the combined disturbance, the energy storage system achieved the strongest overall performance, reducing operating cost, EENS, and peak load shedding by 20.33%, 49.26%, and 30.44%, respectively. Its advantage resulted from discharging during the two critical periods and from enabling greater use of controllable local generation and gas supported conversion. Reconfiguration produced a different benefit: it reduced the number of shedding hours from 19 to 9 and shortened the longest continuous interruption from 19 to 5 h, although its alternative supply paths increased the network loss ratio. Demand response reduced peak shedding by 18.20%, but its energy-neutral structure required the shifted demand to be recovered in other hours. Therefore, storage is more effective when the principal objective is to reduce interruption energy and severity, whereas reconfiguration is more suitable when limiting interruption duration is the dominant operational priority. Demand response provides a lower-cost demand-side option for moderating critical hours, but it cannot replace supply-side energy support. The results also demonstrate that resilience improvement in an integrated energy system should be evaluated across carriers rather than solely from the electrical network perspective. In the storage case, lower electrical curtailment was accompanied by increased gas purchases and greater combined heat and power and fuel cell utilization. This represents an operational substitution from interrupted electrical demand toward dispatchable gas-based conversion, rather than a uniform reduction in all energy and cost components. Similarly, absolute network losses cannot be interpreted independently of served energy because a case with greater load shedding may exhibit lower total losses simply by transferring less electricity. The loss percentage, service restoration trajectory, and cross-carrier dispatch should therefore be assessed together when comparing resilience strategies. The principal conclusions remain consistent across different outage compositions and durations, uncertainty budget levels, voltage requirements, and flexibility cost assumptions. Fixed plan evaluations using samples inside and outside the modeled uncertainty set also preserve the comparative ordering of the event-based cases. All recourse problems were solved successfully, and the numerical audit confirmed the power, thermal, gas, storage, and operating logic constraints, with loss approximation errors remaining below 0.61%. These results indicate that the reported ranking is not dependent on a single disturbance duration, uncertainty setting, or numerical configuration.

Future work can embed the operational framework within a planning model that jointly considers capital expenditure, equipment lifetime, storage degradation, and resource sizing. The formulation can also be extended to coordinate scheduling with time-dependent repair and restoration decisions, progressively revealed operating conditions, and jointly optimized portfolios of reconfiguration, storage, and demand response.

### Limitations of the study

The study has several limitations. The framework is restricted to short-term operational scheduling under prescribed component-availability trajectories and therefore does not optimize repair durations or probabilistic event occurrence. Event probabilities, spatial correlations, and stochastic repair durations are not estimated. Capital expenditure, equipment lifetime, storage degradation, replacement, and resource-sizing decisions are outside the model scope, so the results should not be interpreted as an investment prescription. The flexibility mechanisms are evaluated in separate controlled cases rather than as a jointly optimized portfolio, and the uncertainty treatment uses budgeted adverse-point scheduling rather than an exact min-max robust counterpart or a sequential multistage decision process.

## Resource availability

### Lead contact

Further information and requests for resources should be directed to and will be fulfilled by the lead contact, Majid Moradlou (majid.moradlou@iau.ac.ir).

### Materials availability

This study did not generate new unique materials.

### Data and code availability

• Data: All numerical input data and parameter settings supporting the findings of this study are available from the lead contact upon request. These data have not been deposited in a public repository; therefore, no accession code is applicable. Please refer to the key resources table for additional information.
• Code: The computational code used to implement the proposed framework and generate the reported results is available from the lead contact upon request. The code has not been deposited in a public repository; therefore, no accession code is applicable. Please refer to the key resources table for additional information.
• Additional information: Any additional information required to reanalyze the data reported in this paper is available from the lead contact upon request.

## Acknowledgments

The authors would like to thank their affiliated institutions for providing the academic and technical support required to conduct this research.

## Author contributions

S.E.: conceptualization, methodology, software, validation, formal analysis, investigation, data curation, visualization, writing – original draft, and writing – review and editing. M.M.: supervision, project administration, validation, and writing – review and editing. P.N.: supervision, validation, and writing – review and editing. All authors reviewed and approved the final version of the manuscript.

## Declaration of interests

The authors declare no competing interests.

## Declaration of generative AI and AI-assisted technologies in the writing process

During the preparation of this work, the authors used OpenAI’s ChatGPT to assist with English-language editing and to improve the clarity and readability of the manuscript. After using this tool, the authors critically reviewed, verified, and edited the affected text as needed and take full responsibility for the content of the publication. The tool was not used to generate or analyze simulation data or to replace the authors’ scientific judgment.

## STAR★Methods

### Key resources table

**Table:** 

REAGENT or RESOURCE	SOURCE	IDENTIFIER
GAMS	GAMS Development Corporation	Version 24.1.2
CPLEX solver	IBM	Solver interfaced through GAMS 24.1.2
GAMS source code and model files	This paper	Available from the lead contact upon reasonable request

### Experimental model and study participant details

Omitted as our study does not involve biological models.

### Method details

#### Mathematical formulation

This section formulates the coordinated operation of the electricity-gas-heat distribution system over the scheduling horizon. The electrical network, multi-energy hubs, controllable generators, renewable units, fuel cells, Power-to-Gas (P2G) units, electric and gas boilers, and the case-specific flexibility resources are represented in a single mixed-integer linear framework. Prescribed availability trajectories describe the disturbance pattern, whereas operating uncertainty is confined to electrical demand, thermal demand, wind shortfall, and photovoltaic shortfall.

The solution procedure first identifies an hourly budget-feasible adverse operating point through a support-function linear program. The selected coordinates then enter the scheduling MILP as fixed parameters. Accordingly, the model is classified as budgeted adverse-point scheduling, not as an exact min-max robust counterpart, a two-stage robust program, or an iterative column-and-constraint generation scheme.

The five cases retain the same physical and operational constraints. Case 1 (C1) is the no-event reference, Case 2 (C2) is the predefined combined-outage baseline, Case 3 (C3) enables radial reconfiguration, Case 4 (C4) enables ESS, and Case 5 (C5) enables electrical and thermal demand shifting. The event-type studies are independent reruns under alternative prescribed availability trajectories and do not impose probability weighting or nonanticipativity across events.

##### Predefined disturbance representation

Equation 1 represents the prescribed availability of the upstream connection, generators, wind units, and branches. The disturbance timing and location are fixed before optimization and are not dimensions of the operating uncertainty set. Specifically, Equation 1 bounds the prescribed component-availability factors and fixes branch status when reconfiguration is inactive.

(Equation 1) $$0\leq A_{t}^{\text{UP}},A_{i,t}^{\text{CG}},A_{i,t}^{\text{WT}},A_{ij,t}^{L}\leq 1,s_{ij,t}=A_{ij,t}^{L},\left(i,j\right)\in L\text{.}$$

##### Budgeted adverse operating point

Equations 2, 3, 4, 5, and 6 define the one-sided budgeted uncertainty set and its hourly support problem. Equations 7, 8, 9, 10, 11, 12, and 13 map the selected adverse coordinates to the electrical, thermal, wind, and photovoltaic quantities used by the scheduling model. Specifically, Equation 2 bounds each normalized operating-deviation coordinate; Equation 3 limits the aggregate hourly deviation by the uncertainty budget; Equation 4 links the uncertainty budget to the normalized budget ratio; and Equation 5 selects the hourly adverse coordinate vector through the support-function linear program. Equation 6 defines the engineering stress coefficients used in the initial support problem; Equation 7 applies the selected electrical-demand deviation to active demand; Equation 8 applies the same electrical-demand coordinate to reactive demand and preserves the power-factor relation; and Equation 9 applies the selected thermal-demand deviation. Equation 10 defines wind output before the nonnegativity and unit-availability restrictions; Equation 11 combines wind shortfall with prescribed unit availability and nonnegativity; Equation 12 defines photovoltaic output before the nonnegativity restriction; and Equation 13 enforces nonnegative photovoltaic availability.

(Equation 2) $$0\leq z_{k,t}\leq 1,k\in K\text{.}$$

(Equation 3) $$\sum_{k\in K}z_{k,t}\leq \Gamma \text{.}$$

(Equation 4) Γ=β|K|,0≤β≤1.

(Equation 5) $$\max_{z_{t}\in U_{\Gamma }}\sum_{k\in K}\left(\omega_{k,t}+\varepsilon^{s}\right)z_{k,t},{\hat{z}}_{t}=z_{t}^{⋆}\text{.}$$

(Equation 6) $$\begin{array}{c} \omega_{e,t}^{\text{eng}}=\sum_{i}{\hat{P}}_{i,t}^{D}\text{,}\\ \omega_{\text{th},t}^{\text{eng}}=\frac{\sum_{h}{\hat{T}}_{h,t}^{D}}{\eta^{\text{GB}}}\text{,}\\ \omega_{w,t}^{\text{eng}}=\sum_{i\in W}{\hat{P}}_{i,t}^{\text{WT}}A_{i,t}^{\text{WT}}\text{,}\\ \omega_{\text{pv},t}^{\text{eng}}=\sum_{i\in P}{\hat{P}}_{i,t}^{\text{PV}}\text{.} \end{array}$$

(Equation 7) $$P_{i,t}^{D}={\bar{P}}_{i,t}^{D}+{\hat{z}}_{e,t}{\hat{P}}_{i,t}^{D}\text{.}$$

(Equation 8) $$Q_{i,t}^{D}={\bar{Q}}_{i,t}^{D}+{\hat{z}}_{e,t}{\hat{Q}}_{i,t}^{D}\text{.}$$

(Equation 9) $$T_{h,t}^{D}={\bar{T}}_{h,t}^{D}+{\hat{z}}_{\text{th},t}{\hat{T}}_{h,t}^{D}\text{.}$$

(Equation 10) $${\tilde{P}}_{i,t}^{\text{WT}}={\bar{P}}_{i,t}^{\text{WT}}-{\hat{z}}_{w,t}{\hat{P}}_{i,t}^{\text{WT}}\text{.}$$

(Equation 11) $$P_{i,t}^{\text{WT},\text{av}}=A_{i,t}^{\text{WT}}\;\max\left\{0,{\tilde{P}}_{i,t}^{\text{WT}}\right\}\text{.}$$

(Equation 12) $${\tilde{P}}_{i,t}^{\text{PV}}={\bar{P}}_{i,t}^{\text{PV}}-{\hat{z}}_{\text{pv},t}{\hat{P}}_{i,t}^{\text{PV}}\text{.}$$

(Equation 13) $$P_{i,t}^{\text{PV},\text{av}}=\max\left\{0,{\tilde{P}}_{i,t}^{\text{PV}}\right\}\text{.}$$

##### Operating cost objective and upstream exchange

Equation 14 gives the complete operating-cost objective. Equation 15 constrains upstream active- and reactive-power exchange according to the prescribed grid availability. Specifically, Equation 14 minimizes load-shedding penalties, generation and conversion costs, upstream electricity and gas purchases, transition costs, renewable curtailment, branch-flow regularization, and the case-specific costs of switching, ESS throughput, and demand shifting. Investment and life cycle costs are not included; and Equation 15 imposes availability-dependent limits on upstream active- and reactive-power exchange.

(Equation 14) $$\begin{array}{rr} \min\;Z= & \sum_{i,t}c_{i,t}^{\text{VOLL}}p_{i,t}^{\text{sh}}+\sum_{g,t}\left(c^{G}p_{g,t}^{G}+c_{g,t}^{\text{su},G}+c_{g,t}^{\text{sd},G}\right)\\ & +\sum_{t}c_{t}^{\text{UP}}\left(p_{t}^{\text{imp}}-\rho^{\text{sell}}p_{t}^{\exp}\right)+c^{\text{CHP}}\sum_{h,t}p_{h,t}^{\text{CHP}}\\ & +\sum_{h,t}\left(c_{h,t}^{\text{su},\text{CHP}}+c_{h,t}^{\text{sd},\text{CHP}}\right)+\sum_{h,t}c_{t}^{\text{GW}}G_{h,t}^{\text{GW}}\\ & +\sum_{f,t}\left(c^{\text{FC},\text{OM}}+\frac{c_{t}^{\text{GW}}}{\eta^{\text{FC}}}\right)p_{f,t}^{\text{FC}}+c^{\text{curt}}\sum_{i,t}\left(p_{i,t}^{\text{WT},\text{curt}}+p_{i,t}^{\text{PV},\text{curt}}\right)\\ & +c^{\text{flow}}\sum_{\left(i,j\right)\in L,t}\left(p_{ij,t}^{\text{abs}}+q_{ij,t}^{\text{abs}}\right)+\delta_{3}c^{\text{sw}}\sum_{\left(i,j\right)\in L,t}\Delta u_{ij,t}\\ & +\delta_{4}c^{\deg}\sum_{h,t}\left(p_{h,t}^{\text{ch}}+p_{h,t}^{\text{dis}}\right)\\ & +\delta_{5}\left[c^{\text{DR},E}\sum_{i,t}\left(d_{i,t}^{E,\text{in}}+d_{i,t}^{E,\text{out}}\right)+c^{\text{DR},T}\sum_{h,t}\left(d_{h,t}^{T,\text{in}}+d_{h,t}^{T,\text{out}}\right)\right]\text{.} \end{array}$$

(Equation 15) $$0\leq p_{t}^{\text{imp}},p_{t}^{\exp}\leq {\bar{P}}^{\text{UP}}A_{t}^{\text{UP}}\text{,}-{\bar{Q}}^{\text{UP}}A_{t}^{\text{UP}}\leq q_{t}^{\text{UP}}\leq {\bar{Q}}^{\text{UP}}A_{t}^{\text{UP}}\text{.}$$

##### Electrical network and service constraints

Equations 16, 17, 18, 19, 20, 21, 22, 23, and 24 construct the active-power injections, branch inflows, allocated losses, and nodal active-power balance. Specifically, Equation 16 aggregates the wind and photovoltaic power used at each electrical bus; Equation 17 aggregates controllable-generator and fuel-cell active-power output at each bus; Equation 18 defines the net electrical contribution of the multi-energy hubs; and Equation 19 defines the ESS active-power contribution in the storage-enabled case. Equation 20 combines renewable, firm, hub, and ESS contributions into the local active-power injection; Equation 21 defines the net incoming active branch flow at each bus; Equation 22 allocates one-half of incident active branch losses to each connected bus; and Equation 23 defines net active-power import from the upstream grid. Equation 24 enforces nodal active-power balance.

(Equation 16) $$p_{i,t}^{\text{ren}}=p_{i,t}^{\text{WT},\text{use}}+p_{i,t}^{\text{PV},\text{use}}\text{.}$$

(Equation 17) $$p_{i,t}^{\text{firm}}=\sum_{g\in G_{i}}p_{g,t}^{G}+\sum_{f\in F_{i}}p_{f,t}^{\text{FC}}\text{.}$$

(Equation 18) $$p_{i,t}^{\text{hub}}=\sum_{h\in H_{i}}\left(p_{h,t}^{\text{CHP}}-p_{h,t}^{\text{TB}}-p_{h,t}^{P2G}\right)\text{.}$$

(Equation 19) $$p_{i,t}^{\text{ess}}=\delta_{4}\sum_{h\in H_{i}}\left(p_{h,t}^{\text{dis}}-p_{h,t}^{\text{ch}}\right)\text{.}$$

(Equation 20) $$p_{i,t}^{\text{loc}}=p_{i,t}^{\text{ren}}+p_{i,t}^{\text{firm}}+p_{i,t}^{\text{hub}}+p_{i,t}^{\text{ess}}\text{.}$$

(Equation 21) $$F_{i,t}^{P}=\sum_{j:\left(j,i\right)\in L}P_{ji,t}-\sum_{j:\left(i,j\right)\in L}P_{ij,t}\text{.}$$

(Equation 22) $$L_{i,t}^{P}=\frac{1}{2}\sum_{j:\left\{i,j\right\}\in L}\left(p_{ij,t}^{l}+p_{ji,t}^{l}\right)\text{.}$$

(Equation 23) $$p_{t}^{\text{UP},\text{net}}=p_{t}^{\text{imp}}-p_{t}^{\exp}\text{.}$$

(Equation 24) $$p_{i,t}^{\text{loc}}+r_{i}p_{t}^{\text{UP},\text{net}}+F_{i,t}^{P}=p_{i,t}^{\text{srv}}+L_{i,t}^{P}\text{.}$$

Equations 25, 26, 27, and 28 define the corresponding reactive-power quantities and nodal balance. Specifically, Equation 25 aggregates local reactive sources and upstream reactive support; Equation 26 defines the net incoming reactive branch flow at each bus; Equation 27 allocates one-half of incident reactive branch losses to each connected bus; and Equation 28 enforces nodal reactive-power balance.

(Equation 25) $$q_{i,t}^{\text{src}}=\sum_{g\in G_{i}}q_{g,t}^{G}+\delta_{4}\sum_{h\in H_{i}}q_{h,t}^{\text{ESS}}+r_{i}q_{t}^{\text{UP}}\text{.}$$

(Equation 26) $$F_{i,t}^{Q}=\sum_{j:\left(j,i\right)\in L}Q_{ji,t}-\sum_{j:\left(i,j\right)\in L}Q_{ij,t}\text{.}$$

(Equation 27) $$L_{i,t}^{Q}=\frac{1}{2}\sum_{j:\left\{i,j\right\}\in L}\left(q_{ij,t}^{l}+q_{ji,t}^{l}\right)\text{.}$$

(Equation 28) $$q_{i,t}^{\text{src}}+F_{i,t}^{Q}=q_{i,t}^{\text{srv}}+L_{i,t}^{Q}\text{.}$$

Equations 29, 30, 31, 32, 33, 34, 35, 36, and 37 describe served demand, emergency shedding, reactive-load coupling, and renewable-power allocation. Specifically, Equation 29 defines active demand after the optional electrical shift in C5; Equation 30 links served active demand to adjusted demand and emergency shedding; Equation 31 bounds emergency active-load shedding by the adjusted demand; and Equation 32 defines the adverse reactive-to-active demand ratio when active demand is positive. Equation 33 sets the demand ratio to zero at buses with zero active demand; Equation 34 preserves the adverse power-factor ratio in reactive-load shedding; Equation 35 applies the electrical demand shift to reactive demand in C5; and Equation 36 defines served reactive demand. Equation 37 partitions available wind and photovoltaic power into used and curtailed components.

(Equation 29) $$P_{i,t}^{\text{adj}}=P_{i,t}^{D}+\delta_{5}\left(d_{i,t}^{E,\text{in}}-d_{i,t}^{E,\text{out}}\right)\text{.}$$

(Equation 30) $$p_{i,t}^{\text{srv}}=P_{i,t}^{\text{adj}}-p_{i,t}^{\text{sh}}\text{.}$$

(Equation 31) $$0\leq p_{i,t}^{\text{sh}}\leq P_{i,t}^{\text{adj}}\text{.}$$

(Equation 32) $$\phi_{i,t}P_{i,t}^{D}=Q_{i,t}^{D},P_{i,t}^{D}>0\text{.}$$

(Equation 33) $$\phi_{i,t}=0,P_{i,t}^{D}=0\text{.}$$

(Equation 34) $$q_{i,t}^{\text{sh}}=\phi_{i,t}p_{i,t}^{\text{sh}}\text{.}$$

(Equation 35) $$Q_{i,t}^{\text{adj}}=Q_{i,t}^{D}+\delta_{5}\phi_{i,t}\left(d_{i,t}^{E,\text{in}}-d_{i,t}^{E,\text{out}}\right)\text{.}$$

(Equation 36) $$q_{i,t}^{\text{srv}}=Q_{i,t}^{\text{adj}}-q_{i,t}^{\text{sh}}\text{.}$$

(Equation 37) $$\begin{array}{c} p_{i,t}^{\text{WT},\text{use}}+p_{i,t}^{\text{WT},\text{curt}}=P_{i,t}^{\text{WT},\text{av}}\text{,}\\ p_{i,t}^{\text{PV},\text{use}}+p_{i,t}^{\text{PV},\text{curt}}=P_{i,t}^{\text{PV},\text{av}}\text{.} \end{array}$$

Equations 38, 39, 40, 41, 42, 43, 44, 45, and 46 govern controllable generators and fuel cells, including commitment, availability, transition costs, conversion, and capacity limits. Specifically, Equation 38 couples controllable-generator active output to commitment; Equation 39 couples controllable-generator reactive output to commitment; Equation 40 restricts generator commitment by prescribed availability; and Equation 41 accounts for generator start-up cost at the first hour. Equation 42 sets the first-hour generator shut-down cost to zero; Equation 43 accounts for generator start-up cost after the first hour; Equation 44 accounts for generator shut-down cost after the first hour; and Equation 45 links fuel-cell electrical output to gas input through the conversion efficiency. Equation 46 imposes fuel-cell input and output limits.

(Equation 38) $$0\leq p_{g,t}^{G}\leq {\bar{P}}^{G}u_{g,t}^{G}\text{.}$$

(Equation 39) $$0\leq q_{g,t}^{G}\leq {\bar{Q}}^{G}u_{g,t}^{G}\text{.}$$

(Equation 40) $$0\leq u_{g,t}^{G}\leq A_{g,t}^{\text{CG}}\text{.}$$

(Equation 41) $$c_{g,1}^{\text{su},G}\geq C^{\text{su},G}u_{g,1}^{G}\text{.}$$

(Equation 42) $$c_{g,1}^{\text{sd},G}=0\text{.}$$

(Equation 43) $$c_{g,t}^{\text{su},G}\geq C^{\text{su},G}\left(u_{g,t}^{G}-u_{g,t-1}^{G}\right),t>1\text{.}$$

(Equation 44) $$c_{g,t}^{\text{sd},G}\geq C^{\text{sd},G}\left(u_{g,t-1}^{G}-u_{g,t}^{G}\right),t>1\text{.}$$

(Equation 45) $$p_{f,t}^{\text{FC}}=\eta^{\text{FC}}G_{f,t}^{\text{FC}}\text{.}$$

(Equation 46) $$0\leq p_{f,t}^{\text{FC}}\leq {\bar{P}}_{f}^{\text{FC}},0\leq G_{f,t}^{\text{FC}}\leq \frac{{\bar{P}}_{f}^{\text{FC}}}{\eta^{\text{FC}}}\text{.}$$

##### Multi energy hub constraints

Equations 47, 48, and 49 define adjusted thermal demand and enforce the heat and gas balances of each hub. Specifically, Equation 47 defines thermal demand after the optional thermal shift in C5; Equation 48 enforces the heat balance of each hub; and Equation 49 enforces the gas balance of each hub.

(Equation 47) $$T_{h,t}^{\text{adj}}=T_{h,t}^{D}+\delta_{5}\left(d_{h,t}^{T,\text{in}}-d_{h,t}^{T,\text{out}}\right)\text{.}$$

(Equation 48) $$T_{h,t}^{\text{TB}}+T_{h,t}^{\text{CHP}}+T_{h,t}^{\text{GB}}=T_{h,t}^{\text{adj}}\text{.}$$

(Equation 49) $$G_{h,t}^{\text{GW}}+G_{h,t}^{P2G}=G_{h,t}^{\text{CHP}}+G_{h,t}^{\text{GB}}\text{.}$$

Equations 50, 51, and 52 govern electric-boiler conversion, capacity, and ramping. Specifically, Equation 50 links electric-boiler electrical input to thermal output; Equation 51 imposes the electric-boiler input limit; and Equation 52 limits inter-hour changes in electric-boiler input.

(Equation 50) $$T_{h,t}^{\text{TB}}=\eta^{\text{TB}}p_{h,t}^{\text{TB}}\text{.}$$

(Equation 51) $$0\leq p_{h,t}^{\text{TB}}\leq {\bar{P}}^{\text{TB}}\text{.}$$

(Equation 52) $$-R^{\text{TB}}\leq p_{h,t}^{\text{TB}}-p_{h,t-1}^{\text{TB}}\leq R^{\text{TB}},t>1\text{.}$$

Equations 53, 54, 55, 56, 57, 58, 59, 60, 61, 62, 63, and 64 describe CHP conversion, commitment, ramping, transition costs, and the deterministic thermal-adequacy safeguard used in C2. Specifically, Equation 53 defines the CHP electric, thermal, and combined-efficiency operating region; Equation 54 couples CHP fuel input and useful outputs to commitment; Equation 55 limits inter-hour changes in CHP electrical output; and Equation 56 accounts for CHP start-up cost at the first hour. Equation 57 sets the first-hour CHP shut-down cost to zero; Equation 58 accounts for CHP start-up cost after the first hour; Equation 59 accounts for CHP shut-down cost after the first hour; and Equation 60 defines the upper thermal-demand envelope used by the C2 adequacy safeguard. Equation 61 defines the corresponding nonnegative lower thermal-demand envelope; Equation 62 calculates the aggregate non-CHP thermal capacity; Equation 63 calculates the aggregate adjacent-hour ramp capability of the non-CHP thermal units; and Equation 64 activates the CHP commitment lower bounds associated with thermal capacity and adjacent-hour ramp adequacy.

(Equation 53) $$\begin{array}{c} p_{h,t}^{\text{CHP}}\leq \eta^{\text{CHP},e}G_{h,t}^{\text{CHP}}\text{,}\\ T_{h,t}^{\text{CHP}}\leq \eta^{\text{CHP},h}G_{h,t}^{\text{CHP}}\text{,}\\ p_{h,t}^{\text{CHP}}+T_{h,t}^{\text{CHP}}\leq \eta^{\text{CHP},\text{tot}}G_{h,t}^{\text{CHP}}\text{.} \end{array}$$

(Equation 54) $$\begin{array}{c} 0\leq G_{h,t}^{\text{CHP}}\leq {\bar{G}}^{\text{CHP}}u_{h,t}^{\text{CHP}}\text{,}\\ 0\leq p_{h,t}^{\text{CHP}}\leq {\bar{P}}^{\text{CHP}}u_{h,t}^{\text{CHP}}\text{,}\\ 0\leq T_{h,t}^{\text{CHP}}\leq {\bar{T}}^{\text{CHP}}u_{h,t}^{\text{CHP}}\text{.} \end{array}$$

(Equation 55) $$-R^{\text{CHP}}\leq p_{h,t}^{\text{CHP}}-p_{h,t-1}^{\text{CHP}}\leq R^{\text{CHP}}\text{.}$$

(Equation 56) $$c_{h,1}^{\text{su},\text{CHP}}\geq C^{\text{su},\text{CHP}}u_{h,1}^{\text{CHP}}\text{.}$$

(Equation 57) $$c_{h,1}^{\text{sd},\text{CHP}}=0\text{.}$$

(Equation 58) $$c_{h,t}^{\text{su},\text{CHP}}\geq C^{\text{su},\text{CHP}}\left(u_{h,t}^{\text{CHP}}-u_{h,t-1}^{\text{CHP}}\right)\text{.}$$

(Equation 59) $$c_{h,t}^{\text{sd},\text{CHP}}\geq C^{\text{sd},\text{CHP}}\left(u_{h,t-1}^{\text{CHP}}-u_{h,t}^{\text{CHP}}\right)\text{.}$$

(Equation 60) $$T_{h,t}^{D,U}={\bar{T}}_{h,t}^{D}+{\hat{T}}_{h,t}^{D}\text{.}$$

(Equation 61) $$T_{h,t}^{D,L}=\max\left\{0,{\bar{T}}_{h,t}^{D}-{\hat{T}}_{h,t}^{D}\right\}\text{.}$$

(Equation 62) $$B^{\text{cap}}=\eta^{\text{TB}}{\bar{P}}^{\text{TB}}+\eta^{\text{GB}}{\bar{G}}^{\text{GB}}\text{.}$$

(Equation 63) $$B^{\text{ramp}}=\eta^{\text{TB}}R^{\text{TB}}+\eta^{\text{GB}}R^{\text{GB}}\text{.}$$

(Equation 64) $$\begin{array}{c} u_{h,t}^{\text{CHP}}\geq \kappa_{h,t}^{0}\text{,}\\ u_{h,t}^{\text{CHP}}\geq \kappa_{h,t}^{B}\text{,}\\ u_{h,t}^{\text{CHP}}\geq \kappa_{h,t}^{F}\text{.} \end{array}$$

Equations 65, 66, 67, 68, 69, and 70 govern P2G and gas-boiler conversion, capacity, and ramping. Specifically, Equation 65 links P2G electrical input to gas output; Equation 66 imposes the P2G input limit; Equation 67 limits inter-hour changes in P2G electrical input; and Equation 68 links gas-boiler input to thermal output. Equation 69 imposes the gas-boiler input limit; and Equation 70 limits inter-hour changes in gas-boiler input.

(Equation 65) $$G_{h,t}^{P2G}=\frac{\eta^{P2G}}{\text{HHV}}p_{h,t}^{P2G}\text{.}$$

(Equation 66) $$0\leq p_{h,t}^{P2G}\leq {\bar{P}}^{P2G}\text{.}$$

(Equation 67) $$-R^{P2G}\leq p_{h,t}^{P2G}-p_{h,t-1}^{P2G}\leq R^{P2G},t>1\text{.}$$

(Equation 68) $$T_{h,t}^{\text{GB}}=\eta^{\text{GB}}G_{h,t}^{\text{GB}}\text{.}$$

(Equation 69) $$0\leq G_{h,t}^{\text{GB}}\leq {\bar{G}}^{\text{GB}}\text{.}$$

(Equation 70) $$-R^{\text{GB}}\leq G_{h,t}^{\text{GB}}-G_{h,t-1}^{\text{GB}}\leq R^{\text{GB}},t>1\text{.}$$

##### Branch flow loss and voltage constraints

Equations 71, 72, 73, 74, and 75 define absolute branch flows, the sequential loss approximation, and status-dependent flow and loss limits. Specifically, Equation 71 defines absolute active and reactive branch-flow envelopes and disables them when a branch is unavailable or open; Equation 72 defines the sequential linear approximation of active and reactive branch losses; Equation 73 imposes the status-dependent active branch-flow limit; and Equation 74 imposes the status-dependent reactive branch-flow limit. Equation 75 imposes status-dependent upper bounds on active and reactive branch losses.

(Equation 71) $$\begin{array}{c} p_{ij,t}^{\text{abs}}\geq P_{ij,t},p_{ij,t}^{\text{abs}}\geq -P_{ij,t}\text{,}\\ q_{ij,t}^{\text{abs}}\geq Q_{ij,t},q_{ij,t}^{\text{abs}}\geq -Q_{ij,t}\text{,}\\ 0\leq p_{ij,t}^{\text{abs}},q_{ij,t}^{\text{abs}}\leq \bar{F}\;\sigma_{ij,t}\text{.} \end{array}$$

(Equation 72) $$\begin{array}{c} p_{ij,t}^{l}=\frac{R_{ij}}{1000\;V_{b}^{2}}\left(\lambda_{ij,t}^{P}p_{ij,t}^{\text{abs}}+\lambda_{ij,t}^{Q}q_{ij,t}^{\text{abs}}\right)\text{,}\\ q_{ij,t}^{l}=\frac{X_{ij}}{R_{ij}}p_{ij,t}^{l}\text{.} \end{array}$$

(Equation 73) $$-\bar{F}\sigma_{ij,t}\leq P_{ij,t}\leq \bar{F}\sigma_{ij,t}\text{.}$$

(Equation 74) $$-\bar{F}\sigma_{ij,t}\leq Q_{ij,t}\leq \bar{F}\sigma_{ij,t}\text{.}$$

(Equation 75) $$\begin{array}{c} 0\leq p_{ij,t}^{l}\leq {\bar{L}}^{P}\sigma_{ij,t}\text{,}\\ 0\leq q_{ij,t}^{l}\leq {\bar{L}}^{Q}\sigma_{ij,t}\text{.} \end{array}$$

Equations 76, 77, 78, 79, 80, 81, and 82 enforce the linearized voltage-drop relations, bus-voltage limits, and grid-forming voltage references. Specifically, Equation 76 defines the impedance-dependent voltage-drop term; Equation 77 defines the squared-voltage-drop residual; Equation 78 imposes the upper disjunctive voltage-drop bound; and Equation 79 imposes the lower disjunctive voltage-drop bound. Equation 80 enforces the squared-voltage limits at each bus; Equation 81 fixes the upstream-bus voltage reference; and Equation 82 fixes the grid-forming voltage reference of each supplied island.

(Equation 76) $$\zeta_{ij,t}=\frac{2\left(R_{ij}P_{ij,t}+X_{ij}Q_{ij,t}\right)}{1000V_{b}^{2}}\text{.}$$

(Equation 77) $$\Delta v_{ij,t}=v_{j,t}^{2}-v_{i,t}^{2}+\zeta_{ij,t}\text{.}$$

(Equation 78) $$\Delta v_{ij,t}\leq M^{V}\left(1-\sigma_{ij,t}\right)\text{.}$$

(Equation 79) $$-\Delta v_{ij,t}\leq M^{V}\left(1-\sigma_{ij,t}\right)\text{.}$$

(Equation 80) $${\left({\underline{V}}_{i}\right)}^{2}\leq v_{i,t}^{2}\leq {\left({\bar{V}}_{i}\right)}^{2}\text{.}$$

(Equation 81) $$v_{1,t}^{2}=1\text{.}$$

(Equation 82) $$v_{r,t}^{2}=1,r\in R_{t}^{\text{island}}\text{.}$$

##### Radial network reconfiguration

Equations 83, 84, 85, 86, 87, 88, and 89 impose candidate-line availability, the spanning-tree edge count, and single-commodity connectivity. Specifically, Equation 83 restricts each candidate-line status by its prescribed physical availability; Equation 84 retains the base radial topology at the first hour; Equation 85 imposes the spanning-tree edge count; and Equation 86 injects the fictitious connectivity commodity at the root bus. Equation 87 imposes one unit of fictitious demand at every non-root bus; Equation 88 permits forward connectivity flow only through closed branches; and Equation 89 permits reverse connectivity flow only through closed branches.

(Equation 83) $$0\leq u_{ij,t}^{L}\leq A_{ij,t}^{L},\left(i,j\right)\in L^{c}\text{.}$$

(Equation 84) $$u_{ij,1}^{L}=u_{ij}^{0},\left(i,j\right)\in L^{c}\text{.}$$

(Equation 85) $$\sum_{\left(i,j\right)\in L^{c}}u_{ij,t}^{L}=\left|B\right|-1\text{.}$$

(Equation 86) $$\sum_{j}f_{1j,t}^{c}-\sum_{j}f_{j1,t}^{c}=\left|B\right|-1\text{.}$$

(Equation 87) $$\sum_{j}f_{ji,t}^{c}-\sum_{j}f_{ij,t}^{c}=1,i\neq 1\text{.}$$

(Equation 88) $$0\leq f_{ij,t}^{c}\leq \left(\left|B\right|-1\right)u_{ij,t}^{L}\text{.}$$

(Equation 89) $$0\leq f_{ji,t}^{c}\leq \left(\left|B\right|-1\right)u_{ij,t}^{L}\text{.}$$

Equations 90, 91, 92, 93, 94, 95, 96, 97, 98, 99, and 100 count switching actions and restrict topology changes to eligible availability transitions. Specifically, Equation 90 lower-bounds the first-hour switching indicator for line closure; Equation 91 lower-bounds the first-hour switching indicator for line opening; Equation 92 lower-bounds subsequent switching indicators for line closure; and Equation 93 lower-bounds subsequent switching indicators for line opening. Equation 94 limits the number of switching actions at each eligible transition; Equation 95 identifies hours at which prescribed component availability changes; Equation 96 aggregates the availability changes of the upstream grid, generators, wind units, and lines; and Equation 97 measures the change in upstream-grid availability. Equation 98 aggregates controllable-generator availability changes; Equation 99 aggregates wind-unit availability changes; and Equation 100 aggregates branch-availability changes.

(Equation 90) $$\Delta u_{ij,1}\geq u_{ij,1}^{L}-u_{ij}^{0}\text{.}$$

(Equation 91) $$\Delta u_{ij,1}\geq u_{ij}^{0}-u_{ij,1}^{L}\text{.}$$

(Equation 92) $$\Delta u_{ij,t}\geq u_{ij,t}^{L}-u_{ij,t-1}^{L},t>1\text{.}$$

(Equation 93) $$\Delta u_{ij,t}\geq u_{ij,t-1}^{L}-u_{ij,t}^{L},t>1\text{.}$$

(Equation 94) $$\sum_{\left(i,j\right)\in L^{c}}\Delta u_{ij,t}\leq N^{\text{sw},\max}\chi_{t}\text{.}$$

(Equation 95) $$\chi_{t}=I\left(\Delta A_{t}>0\right),t>1\text{.}$$

(Equation 96) $$\Delta A_{t}=\Delta A_{t}^{\text{UP}}+\Delta A_{t}^{\text{CG}}+\Delta A_{t}^{\text{WT}}+\Delta A_{t}^{L}\text{.}$$

(Equation 97) $$\Delta A_{t}^{\text{UP}}=\left|A_{t}^{\text{UP}}-A_{t-1}^{\text{UP}}\right|\text{.}$$

(Equation 98) $$\Delta A_{t}^{\text{CG}}=\sum_{g}\left|A_{g,t}^{\text{CG}}-A_{g,t-1}^{\text{CG}}\right|\text{.}$$

(Equation 99) $$\Delta A_{t}^{\text{WT}}=\sum_{i}\left|A_{i,t}^{\text{WT}}-A_{i,t-1}^{\text{WT}}\right|\text{.}$$

(Equation 100) $$\Delta A_{t}^{L}=\sum_{\left(i,j\right)\in L^{c}}\left|A_{ij,t}^{L}-A_{ij,t-1}^{L}\right|\text{.}$$

##### ESS and demand response

Equations 101, 102, 103, 104, 105, and 106 govern ESS energy evolution, terminal inventory, charging and discharging power, and reactive support. Equations 107, 108, and 109 impose energy-neutral electrical and thermal demand shifting and their hourly limits. Specifically, Equation 101 defines the first-hour ESS energy state; Equation 102 defines the ESS energy recursion for subsequent hours; Equation 103 enforces the ESS energy bounds; and Equation 104 restores the initial ESS inventory at the end of the horizon. Equation 105 imposes nonnegative charging and discharging power and their combined converter limit; Equation 106 imposes the ESS reactive-power capability limits; Equation 107 enforces energy neutrality of the electrical demand shift at each bus; and Equation 108 enforces energy neutrality of the thermal demand shift at each hub. Equation 109 limits hourly electrical and thermal shifting by the admissible demand fractions.

(Equation 101) $$\begin{array}{c} E_{h,1}^{\text{ESS}}=E^{\text{ini}}+\Delta t\;\eta^{\text{ESS}}p_{h,1}^{\text{ch}} \end{array}-\Delta t\;\frac{p_{h,1}^{\text{dis}}}{\eta^{\text{ESS}}}\text{.}$$

(Equation 102) $$\begin{array}{c} E_{h,t}^{\text{ESS}}=E_{h,t-1}^{\text{ESS}}+\Delta t\;\eta^{\text{ESS}}p_{h,t}^{\text{ch}}-\Delta t\;\frac{p_{h,t}^{\text{dis}}}{\eta^{\text{ESS}}},t>1\text{.} \end{array}$$

(Equation 103) $$0\leq E_{h,t}^{\text{ESS}}\leq {\bar{E}}^{\text{ESS}}\text{.}$$

(Equation 104) $$E_{h,T}^{\text{ESS}}=E^{\text{ini}}\text{.}$$

(Equation 105) $$0\leq p_{h,t}^{\text{ch}},0\leq p_{h,t}^{\text{dis}},p_{h,t}^{\text{ch}}+p_{h,t}^{\text{dis}}\leq {\bar{P}}^{\text{ESS}}\text{.}$$

(Equation 106) $${\underline{Q}}^{\text{ESS}}\leq q_{h,t}^{\text{ESS}}\leq {\bar{Q}}^{\text{ESS}}\text{.}$$

(Equation 107) $$\sum_{t}d_{i,t}^{E,\text{in}}=\sum_{t}d_{i,t}^{E,\text{out}},\forall i\text{.}$$

(Equation 108) $$\sum_{t}d_{h,t}^{T,\text{in}}=\sum_{t}d_{h,t}^{T,\text{out}},\forall h\text{.}$$

(Equation 109) $$\begin{array}{c} 0\leq d_{i,t}^{E,\text{in}},d_{i,t}^{E,\text{out}}\leq \psi^{\text{DR}}{\bar{P}}_{i,t}^{D}\text{,}\\ 0\leq d_{h,t}^{T,\text{in}},d_{h,t}^{T,\text{out}}\leq \psi^{\text{DR}}{\bar{T}}_{h,t}^{D}\text{.} \end{array}$$

##### Resilience interruption and recovery metrics

Equations 110, 111, 112, 113, 114, 115, 116, 117, 118, 119, and 120 define hourly and energy-based resilience indices, EENS, the average resilience measure, and the resilience nadir. EENS quantifies the total electrical energy demand that cannot be served during the study period due to system disturbances or operational limitations.^29^ Specifically, Equation 110 aggregates bus-level active-load shedding at each hour; Equation 111 removes numerical shedding values below the reporting tolerance; Equation 112 aggregates the adjusted electrical demand at each hour; and Equation 113 defines the hourly RI from screened shedding and adjusted demand. Equation 114 specifies the numerical threshold used to identify full service; Equation 115 calculates EENS over the scheduling horizon; Equation 116 calculates total adjusted electrical-demand energy; and Equation 117 defines the energy-based RI. Equation 118 defines the horizon-average RI; Equation 119 identifies the minimum hourly RI; and Equation 120 identifies the hour of the resilience nadir.

(Equation 110) $$H_{t}^{\text{raw}}=\sum_{i}p_{i,t}^{\text{sh}}\text{.}$$

(Equation 111) $$H_{t}=H_{t}^{\text{raw}}\;I\left(H_{t}^{\text{raw}}>\varepsilon^{P}\right)\text{.}$$

(Equation 112) $$D_{t}=\sum_{i}P_{i,t}^{\text{adj}}\text{.}$$

(Equation 113) $$RI_{t}=100\left(1-\frac{H_{t}}{\max\left\{\varepsilon ,D_{t}\right\}}\right)\text{.}$$

(Equation 114) $$RI^{\text{full}}=100-\varepsilon^{RI}\text{.}$$

(Equation 115) $$\text{EENS}=\Delta t\sum_{t}H_{t}\text{.}$$

(Equation 116) $$E^{D}=\Delta t\sum_{t}D_{t}\text{.}$$

(Equation 117) $$RI^{E}=100\left(1-\frac{\text{EENS}}{E^{D}}\right)\text{.}$$

(Equation 118) $$RI^{\text{AUC}}=\frac{1}{\left|T\right|}\sum_{t}RI_{t}\text{.}$$

(Equation 119) $$RI^{\text{nad}}=\min_{t}RI_{t}\text{.}$$

(Equation 120) $$t^{\text{nad}}=\min\left\{t:RI_{t}=RI^{\text{nad}}\right\}\text{.}$$

Equations 121, 122, 123, 124, 125, 126, 127, 128, 129, 130, 131, 132, 133, 134, and 135 quantify interruption duration, event timing, service recovery, recovery slope, residual energy deficit, and bus-level resilience. Specifically, Equation 121 identifies hours with material load shedding; Equation 122 counts the hours with load shedding; Equation 123 tracks the running duration of each uninterrupted shedding interval; and Equation 124 identifies the longest uninterrupted shedding interval. Equation 125 determines the minimum availability across monitored components at each hour; Equation 126 identifies hours containing at least one prescribed component outage; Equation 127 identifies the final hour of the prescribed event; and Equation 128 identifies the first full-service hour after the event. Equation 129 calculates the recovery time measured from the end of the event; Equation 130 identifies the first full-service hour after the resilience nadir; Equation 131 calculates the recovery time measured from the nadir; and Equation 132 calculates the average recovery slope from the nadir to full service. Equation 133 calculates the accumulated energy deficit from the nadir onward; Equation 134 calculates bus-level EENS; and Equation 135 defines the bus-level energy-based RI.

(Equation 121) $$y_{t}=I\left(H_{t}>0\right)\text{.}$$

(Equation 122) $$N^{\text{sh}}=\sum_{t}y_{t}\text{.}$$

(Equation 123) $$d_{t}=y_{t}\left(d_{t-1}+1\right),d_{0}=0\text{.}$$

(Equation 124) $$D^{\text{long}}=\max_{t}d_{t}\text{.}$$

(Equation 125) $$a_{t}^{\min}=\min_{c\in C}A_{c,t}\text{.}$$

(Equation 126) $$o_{t}=I\left(a_{t}^{\min}<1\right)\text{.}$$

(Equation 127) $$t^{e}=\max\left\{t:o_{t}=1\right\}\text{.}$$

(Equation 128) $$t^{\text{rec},e}=\min\left\{t>t^{e}:RI_{t}\geq RI^{\text{full}}\right\}\text{.}$$

(Equation 129) $$\tau^{\text{rec},e}=t^{\text{rec},e}-t^{e}\text{.}$$

(Equation 130) $$t^{\text{rec},n}=\min\left\{t>t^{\text{nad}}:RI_{t}\geq RI^{\text{full}}\right\}\text{.}$$

(Equation 131) $$\tau^{\text{rec},n}=t^{\text{rec},n}-t^{\text{nad}}\text{.}$$

(Equation 132) $$s^{\text{rec}}=\frac{100-RI^{\text{nad}}}{\tau^{\text{rec},n}}\text{.}$$

(Equation 133) $$E^{\mathrm{def},n}=\Delta t\sum_{t\geq t^{\text{nad}}}H_{t}\text{.}$$

(Equation 134) $${\text{EENS}}_{i}=\Delta t\sum_{t}p_{i,t}^{\text{sh}}\text{.}$$

(Equation 135) $$RI_{i}=100\left(1-\frac{{\text{EENS}}_{i}}{\Delta t\sum_{t}P_{i,t}^{D}}\right)\text{.}$$

##### Loss refinement and dual guided adverse point update

Equations 136 and 137 evaluate the exact post-solution branch losses and the error of the sequential approximation. Equations 138, 139, 140, and 141 update the adverse-point support coefficients using the relaxed-model marginal values. Specifically, Equation 136 evaluates active and reactive branch losses from the optimized flows in the ex-post accuracy check; Equation 137 measures the relative error of the sequential active-loss approximation; Equation 138 updates the electrical-demand support coefficient using relaxed-model marginal values; and Equation 139 updates the thermal-demand support coefficient. Equation 140 updates the wind-shortfall support coefficient; and Equation 141 updates the photovoltaic-shortfall support coefficient.

(Equation 136) $${\tilde{p}}_{ij,t}^{l}=\frac{R_{ij}\left(P_{ij,t}^{2}+Q_{ij,t}^{2}\right)}{1000\;V_{b}^{2}},{\tilde{q}}_{ij,t}^{l}=\frac{X_{ij}}{R_{ij}}{\tilde{p}}_{ij,t}^{l}\text{.}$$

(Equation 137) $$\varepsilon_{\%}^{l}=100\;\frac{\left|\Delta t\sum_{\left(i,j\right),t}p_{ij,t}^{l}-\Delta t\sum_{\left(i,j\right),t}{\tilde{p}}_{ij,t}^{l}\right|}{\max\left\{\varepsilon ,\Delta t\sum_{\left(i,j\right),t}{\tilde{p}}_{ij,t}^{l}\right\}}\text{.}$$

(Equation 138) $$\omega_{e,t}^{\text{dual}}=\sum_{i}\left(\left|\pi_{i,t}^{P}\right|{\hat{P}}_{i,t}^{D}+\left|\pi_{i,t}^{Q}\right|{\hat{Q}}_{i,t}^{D}\right)+\varepsilon^{\omega }\omega_{e,t}^{\text{eng}}\text{.}$$

(Equation 139) $$\omega_{\text{th},t}^{\text{dual}}=\sum_{h}\left|\pi_{h,t}^{T}\right|{\hat{T}}_{h,t}^{D}+\varepsilon^{\omega }\omega_{\text{th},t}^{\text{eng}}\text{.}$$

(Equation 140) $$\omega_{w,t}^{\text{dual}}=\sum_{i\in W}\left|\pi_{i,t}^{W}\right|{\hat{P}}_{i,t}^{\text{WT}}A_{i,t}^{\text{WT}}+\varepsilon^{\omega }\omega_{w,t}^{\text{eng}}\text{.}$$

(Equation 141) $$\omega_{\text{pv},t}^{\text{dual}}=\sum_{i\in P}\left|\pi_{i,t}^{PV}\right|{\hat{P}}_{i,t}^{\text{PV}}+\varepsilon^{\omega }\omega_{\text{pv},t}^{\text{eng}}\text{.}$$

The computational sequence proceeds as follows:

First, the hourly support LP is solved using nonnegative engineering stress coefficients to obtain an initial budget-feasible adverse operating point. Second, the scheduling MILP is solved through case-specific sequential updates of the linear loss-reference factors. Third, the validated unit-commitment decisions and, in C3, the branch-status schedule are temporarily fixed, and the relaxed scheduling model is solved once to obtain marginal information. These marginal values update the four support coefficients associated with electrical demand, thermal demand, wind shortfall, and photovoltaic shortfall. The hourly support LPs are then resolved, and the adverse coordinates are updated when their maximum change exceeds 10^−6^. Finally, integrality is restored, the scheduling MILP is resolved, and additional loss-reference updates are performed only to satisfy the post-solution loss-accuracy criterion. This procedure contains one dual-guided screening pass and does not constitute an iterative adversarial min–max or column-and-constraint generation algorithm.

##### Fixed plan sensitivity and OOS stress validation

Equations 142, 143, 144, and 145 generate in-set and deliberately outside-set operating realizations. Equations 146, 147, 148, and 149 define fixed-plan continuous recourse and the in-set dominance checks for cost and EENS. Specifically, Equation 142 generates preliminary coordinates for in-set stress samples; Equation 143 radially projects samples that exceed the design uncertainty budget; Equation 144 generates the auxiliary random draws used for the outside-set stress group; and Equation 145 places the outside-set coordinates beyond the design set when the budget is binding. Equation 146 minimizes the operating cost of each fixed-plan recourse problem; Equation 147 defines the feasible continuous-recourse set for the retained case-specific day-ahead plan; Equation 148 states the in-set cost-dominance check relative to the design adverse point; and Equation 149 states the corresponding in-set EENS-dominance check.

(Equation 142) $$\xi_{n,t,k}∼U\left(0,1\right)\text{.}$$

(Equation 143) $$\xi_{n,t,k}^{\prime }=\xi_{n,t,k}\;\min\left\{1,\frac{\Gamma }{\sum_{k^{\prime }}\xi_{n,t,k^{\prime }}}\right\}\text{.}$$

(Equation 144) $$U_{n,t,k}∼U\left(0,1\right)\text{.}$$

(Equation 145) $$\xi_{n,t,k}^{\text{out}}=0.625+0.125U_{n,t,k},\Gamma <\left|K\right|\text{.}$$

(Equation 146) $$Z_{n}^{\text{rec}}=\min_{y_{n}}C\left(\bar{x},y_{n};\xi_{n},A\right)\text{.}$$

(Equation 147) $$y_{n}\in Y\left(\bar{x},\xi_{n},A\right)\text{.}$$

(Equation 148) $$Z_{n}^{\text{rec}}\leq Z^{\text{adv}}+\varepsilon^{C},n\in N^{\text{in}}\text{.}$$

(Equation 149) $${\text{EENS}}_{n}\leq {\text{EENS}}^{\text{adv}}+\varepsilon^{E},n\in N^{\text{in}}\text{.}$$

The retained plan is defined separately for each case. In C2, the controllable-generator and CHP commitment schedules are fixed. In C3, the generator and CHP commitments and the hourly branch-status schedule are fixed. In C4, the commitments and the ESS charging, discharging, and reactive-support schedules are fixed. In C5, the commitments and the electrical and thermal demand-shifting schedules are fixed. Continuous redispatch of generation, energy conversion, upstream exchange, network flows, renewable curtailment, and emergency load shedding remains available in the recourse problems. Consequently, the validation assesses the operational adaptability of the retained plan and does not perform a new full day-ahead optimization for each realization.

##### Compact formulation and model classification

Equations 150, 151, 152, and 153 summarize the sequential support problem, scheduling objective, operating constraints, and decision domains. Specifically, Equation 150 gives the compact hourly support problem; Equation 151 gives the compact scheduling objective; Equation 152 represents the compact operating constraints; and Equation 153 specifies the binary and continuous decision domains.

(Equation 150) $${\hat{z}}_{t}\in \arg\max_{z_{t}\in U_{\Gamma }}\omega_{t}^{⊤}z_{t}\text{.}$$

(Equation 151) $$\min_{x,y}C\left(x,y\right)\text{.}$$

(Equation 152) $$\text{Ax}+\text{By}\geq b\left(\hat{z},a\right)\text{.}$$

(Equation 153) $$x\in {\left\{0,1\right\}}^{n_{b}},y\in R^{n_{c}}\text{.}$$

For fixed loss-reference factors, the scheduling problem is linear. The binary variables represent generator and CHP commitment and, in C3, candidate-branch status; the remaining operating quantities are continuous. The support problem is an LP, whereas the scheduling and loss-refinement problems are MILPs. The exact quadratic loss expression is used only in the post-solution accuracy check and does not enter branch-and-bound.

The operational scheduling model is therefore single stage with respect to the decisions made at the selected adverse operating point. The support LP is a deterministic preprocessing problem rather than the second-stage adversarial subproblem of a two-stage robust formulation. The single relaxed-model solve used to update the support coefficients and the sequential loss-reference updates are computational refinement steps and do not convert the formulation into a two-stage robust program.

#### Test system input data and simulation setup

This section describes the test system and the numerical data used in the case studies. The scheduling horizon comprises 24 1-h intervals. The network, resource locations, demand data, prices, and uncertainty settings are unchanged across the five cases; the cases differ only in the prescribed disturbance pattern and the enabled flexibility mechanism.

##### Integrated system configuration

**Figure 10:**
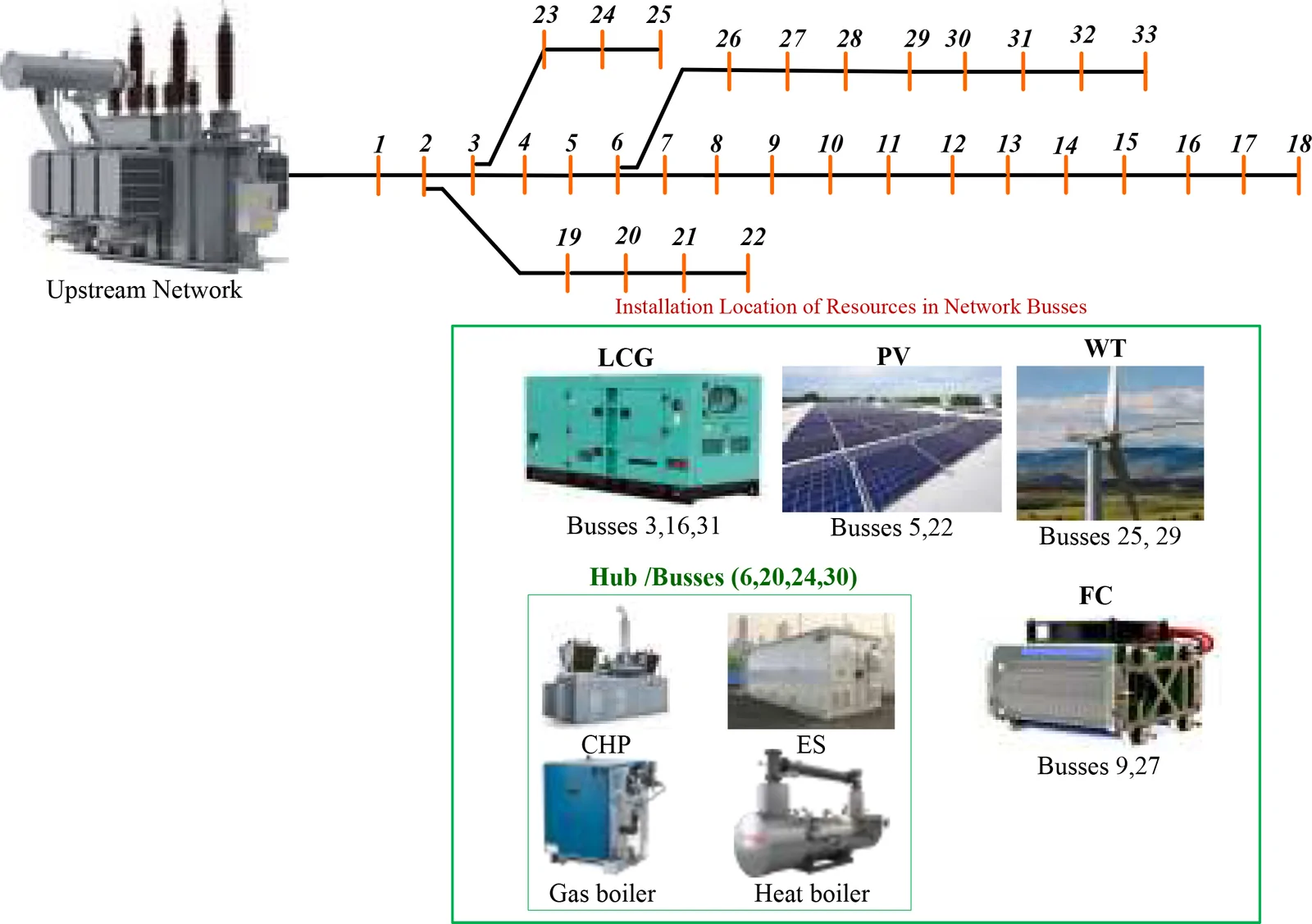
Modified IEEE 33-bus distribution system and locations of controllable generators, photovoltaic units, wind units, fuel cells, and multi-energy hubs

The electrical layer is based on the 12.66-kV IEEE 33-bus radial distribution feeder. The base active and reactive demands are 3715 kW and 1800 kVAr, respectively. Multi-energy hubs are connected at buses 6, 20, 24, and 30. The active demand at these four buses is multiplied by 2 before applying the hourly demand profile, while the base thermal demands are 450, 400, 350, and 550 kW, respectively. Figure 10 shows the feeder structure and the locations of the electrical and multi-energy resources.

To provide a clear overview of the resource configuration used in the simulations, the following resource-location table summarizes the bus locations, number of installed units, nominal ratings, and relevant case-specific settings of the electrical and multi-energy resources. The system includes controllable generators, photovoltaic and wind units, fuel cells, multi-energy hubs, electrical storage units, and electrical and thermal demand response. The underlying resource locations and technical ratings remain unchanged across the case studies, while the availability of selected components and the activation of ESS and DR depend on the considered case.

#### Resource locations and nominal ratings

**Table:** 

Resource	Bus location	Number	Main rating	Additional setting
Controllable generator	3, 16, 31	3	*P*^G,*max*^,*Q*^G,*max*^	500 kW; 250 kVAr
Photovoltaic unit	5, 22	2	250 kW/unit	Hourly profile in Figure 12
Wind unit	25, 29	2	400 kW/unit	Bus 29 unavailable in C2–C5
Fuel cell	9, 27	2	$P_{f}^{\text{FC},\max}$	200 kW; efficiency 0.50
Multi-energy hub	6, 20, 24, 30	4	CHP, TB, GB, P2G	Thermal demands: 450, 400, 350, 550 kW
Electrical storage (C4)	6, 20, 24, 30	4	*E*^*max*^	600 kWh; 150 kW per hub
DR (C5)	All buses/hubs	–	*ψ*^DR^	15% electrical and thermal shifting limit

The following table lists the bus-level demand data used to construct the hourly nominal quantities in the mathematical formulation. The first hourly profile, denoted p1 in the GAMS input, is the only load multiplier used by the final model. The table is arranged in two blocks to reduce the required space.

#### Base electrical and thermal demand data

**Table:** 

Bus	P (kW)	Q (kVAr)	T (kW)	Bus	P (kW)	Q (kVAr)	T (kW)
1	0	0	0	18	90	40	0
2	100	60	0	19	90	40	0
3	90	40	0	20	90	40	400
4	120	80	0	21	90	40	0
5	60	30	0	22	90	40	0
6	60	20	450	23	90	50	0
7	200	100	0	24	420	200	350
8	200	100	0	25	420	200	0
9	60	20	0	26	60	25	0
10	60	20	0	27	60	25	0
11	45	30	0	28	60	20	0
12	60	35	0	29	120	70	0
13	60	35	0	30	200	100	550
14	120	80	0	31	150	70	0
15	60	10	0	32	210	100	0
16	60	20	0	33	60	40	0
17	60	20	0	–	–	–	–

##### Multi energy conversion and flexibility data

Each hub contains a CHP unit, an electric boiler, a gas boiler, and a P2G unit. The following table summarizes the corresponding capacity, efficiency, and ramp-rate data. The CHP operating domain is represented directly by the electrical, thermal, and combined useful-efficiency limits in Equation 53, while commitment and output bounds are enforced by Equation 54. The remaining conversion limits and ramp constraints are applied through Equations 50, 51, 52, and 65, 66, 67, 68, 69, 70.

#### Technical data of the multi-energy conversion and flexibility resources

**Table:** 

Component	Parameter	Value	Unit/note
CHP	*P*^CHP,*max*^	400	kW
CHP	*T*^CHP,*max*^	350	kW
CHP	*G*^CHP,*max*^	1000	kW-eq
CHP	*η*^CHP,e^	0.40	–
CHP	*η*^CHP,h^	0.50	–
CHP	*η*^CHP,tot^	0.80	–
CHP	*R*^CHP^	120	kW/h
Electric boiler	*P*^TB,*max*^	250	kW
Electric boiler	*η*^TB^	0.90	–
Electric boiler	*R*^TB^	25	kW/h
Gas boiler	*G*^GB,*max*^	250	kW-eq
Gas boiler	*η*^GB^	0.90	–
P2G	*P*^P2G,*max*^	250	kW
P2G	*η*^P2G^	0.90	–

In C4, the storage units have an initial and terminal energy of 50 kWh, an energy capacity of 600 kWh, a shared charging/discharging limit of 150 kW, a reactive-power range of −100 to 100 kVAr, and a charging/discharging efficiency of 0.90. In C5, electrical and thermal demand shifting is energy neutral over the 24-h horizon and is limited to 15% of the corresponding nominal demand in each direction.

##### Distribution network and reconfiguration setting

**Figure 11:**
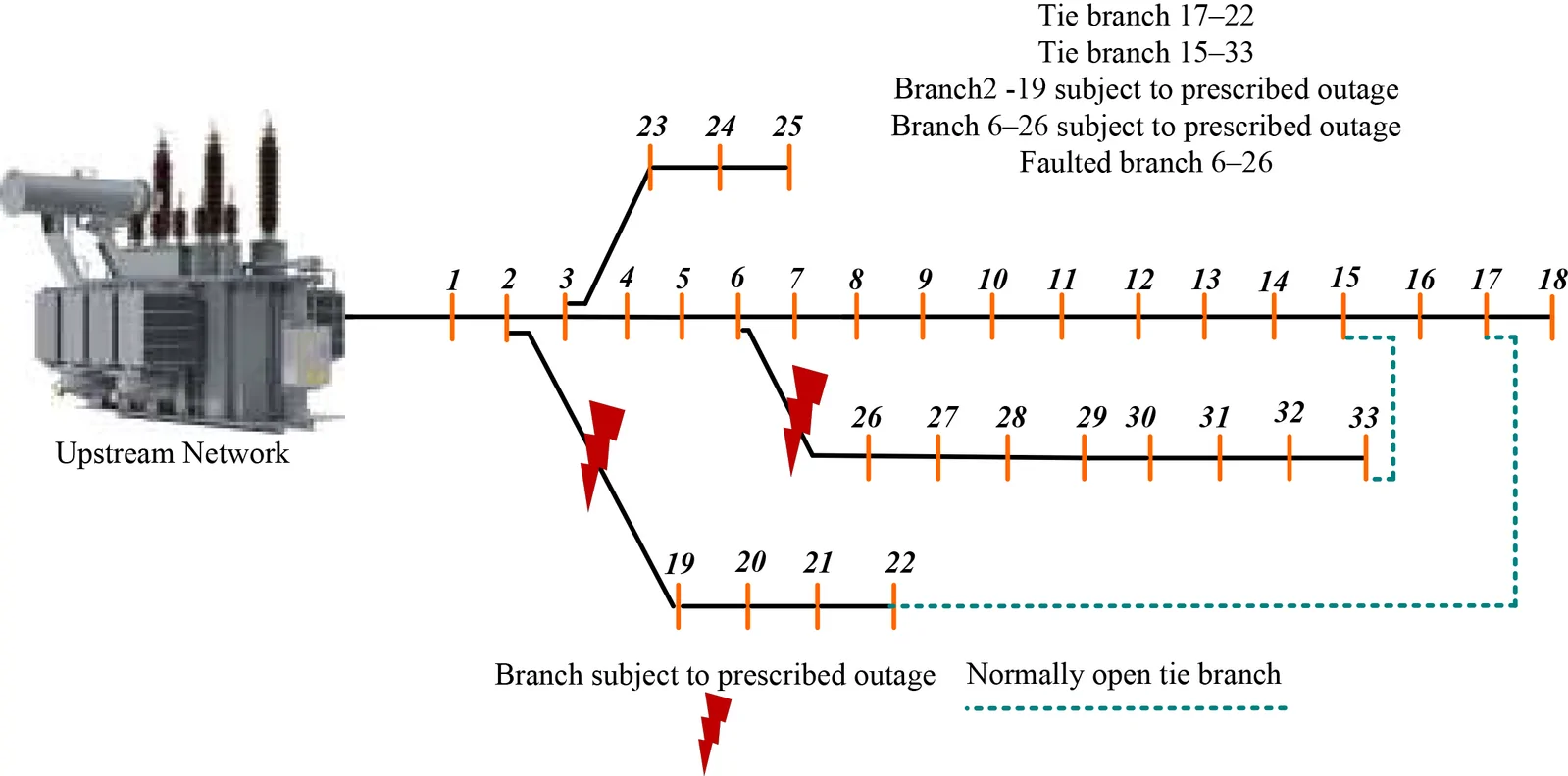
Base radial feeder and candidate switching branches used in the reconfiguration case

Following an extreme event, timely and effective network reconfiguration is essential to restore feasible power delivery paths and limit service interruptions.^30^ Figure 11 presents the candidate network used by the reconfiguration model. The network consists of the 32 branches of the base IEEE 33-bus feeder and two normally open tie branches, 15–33 and 17–22. All base branches are represented as switchable sectionalizing branches. At each hour, the model retains 32 energized branches and enforces connectivity and radiality. The availability of branches 2–19 and 6–26 is restricted during their prescribed outage intervals, while topology changes are limited to the hours at which the component-availability pattern changes.

The following table presents the resistance and reactance data of the base feeder branches and the two normally open tie branches used for network reconfiguration.

#### Branch resistance and reactance data; tie branches are marked explicitly

**Table:** 

Branch	R (Ω)	X (Ω)	Type	Branch	R (Ω)	X (Ω)	Type
1–2	0.0922	0.0470	Base	15–16	0.7463	0.5450	Base
2–3	0.4930	0.2511	Base	16–17	1.2890	1.7210	Base
2–19	0.1640	0.1565	Base	17–18	0.7320	0.5740	Base
3–4	0.3660	0.1864	Base	19–20	1.5042	1.3554	Base
3–23	0.4512	0.3083	Base	20–21	0.4095	0.4784	Base
4–5	0.3811	0.1941	Base	21–22	0.7089	0.9373	Base
5–6	0.8190	0.7070	Base	23–24	0.8980	0.7091	Base
6–7	0.1872	0.6188	Base	24–25	0.8960	0.7011	Base
6–26	0.2030	0.1034	Base	26–27	0.2842	0.1447	Base
7–8	0.7114	0.2351	Base	27–28	1.0590	0.9337	Base
8–9	1.0300	0.7400	Base	28–29	0.8042	0.7006	Base
9–10	1.0440	0.7400	Base	29–30	0.5075	0.2585	Base
10–11	0.1966	0.0650	Base	30–31	0.9744	0.9630	Base
11–12	0.3744	0.1238	Base	31–32	0.3105	0.3619	Base
12–13	1.4680	1.1550	Base	32–33	0.3410	0.5302	Base
13–14	0.5416	0.7129	Base	17–22	0.1000	0.0700	Tie
14–15	0.5910	0.5260	Base	15–33	0.1000	0.0700	Tie

The following table summarizes the main electrical network limits and switching parameters applied in the simulations.

#### Network and reconfiguration parameters

**Table:** 

Parameter	Symbol	Value	Unit/note
Base voltage	*V*_b_	12.66	kV
Voltage limits	$V_{i}^{\min},V_{i}^{\max}$	0.90–1.10	p.u.
Upstream limits	*P*^UP,*max*^,*Q*^UP,*max*^	3000/2000	kW/kVAr
Branch-flow limit	*P*^flow,*max*^	6000	kW and kVAr
Active-loss limit	*P*^loss,*max*^	1000	kW
Switching limit	*N*^sw,*max*^	6	actions per permitted transition
Switching cost	*c*^sw^	0.02	US$/action

#### Prescribed disturbances and operating uncertainty

The combined disturbance used in C2–C5 contains upstream-grid disconnections, the loss of controllable generator 3, the full-day unavailability of the wind unit at bus 29, and outages of branches 6–26 and 2–19. These events are fixed before optimization and are not included in the operating uncertainty set. The following table gives their locations and durations.

#### Prescribed component outages in the combined event

**Table:** 

Unavailable component	Availability parameter	Location	Outage hours
Upstream grid	$A_{t}^{\text{UP}}$	Root connection	9–12; 18–22
Wind unit	$A_{i,t}^{\text{WT}}$	Bus 29	1–24
Controllable generator	$A_{i,t}^{\text{CG}}$	Bus 3	3–9; 14–19
Distribution branch	$A_{ij,t}^{L}$	2–19	13–23
Distribution branch	$A_{ij,t}^{L}$	6–26	5–15

When branch 2–19 is unavailable, buses 19–22 form a separated area. The CHP at bus 20 provides the voltage reference in C2 and C5, while the storage inverter at bus 20 serves this role in C4. The outage of branch 6–26 separates buses 26–33; the controllable generator at bus 31 provides the reference in C2 and C5, and the storage inverter at bus 30 provides it in C4. In C3, the candidate tie branches allow the radial topology to be restored without retaining these two islands. The prescribed event set is used as an engineering stress test. The upstream-grid disconnections represent temporary islanding conditions. The outage of branch 2–19 separates buses 19–22, while the outage of branch 6–26 separates buses 26–33; these cases examine whether local grid-forming resources or radial reconfiguration can maintain supply to the separated areas. The outage of controllable generator 3 represents the loss of local dispatchable generation, whereas the full-day unavailability of wind unit 29 represents the loss of a renewable resource. The availability trajectories include overlapping and non-overlapping outage intervals to examine isolated and combined supply shortages. They are prescribed operating scenarios rather than probabilistic hazard realizations. Event probabilities, spatial correlations, and stochastic repair durations are not estimated in this study.

Operating uncertainty covers electrical demand, thermal demand, wind-generation shortfall, and photovoltaic-generation shortfall. Each nominal profile is assigned a 10% one-sided deviation. With four uncertainty dimensions, β = 0.60 yields an hourly uncertainty budget of Γ = 2.40. The value β = 0.60 is used as an intermediate budget level, allowing the support problem to distribute the available budget across multiple deviations without imposing either the nominal condition (β = 0) or the simultaneous full deviation of all four dimensions (β = 1). Reactive demand follows the electrical-demand coordinate to preserve the active and reactive demand relationship. Section 2.6 examines β ∈ {0,0.4,0.6,1.0} to evaluate the dependence of the results on this selection.

##### Time varying inputs cost coefficients and computational settings

**Figure 12:**
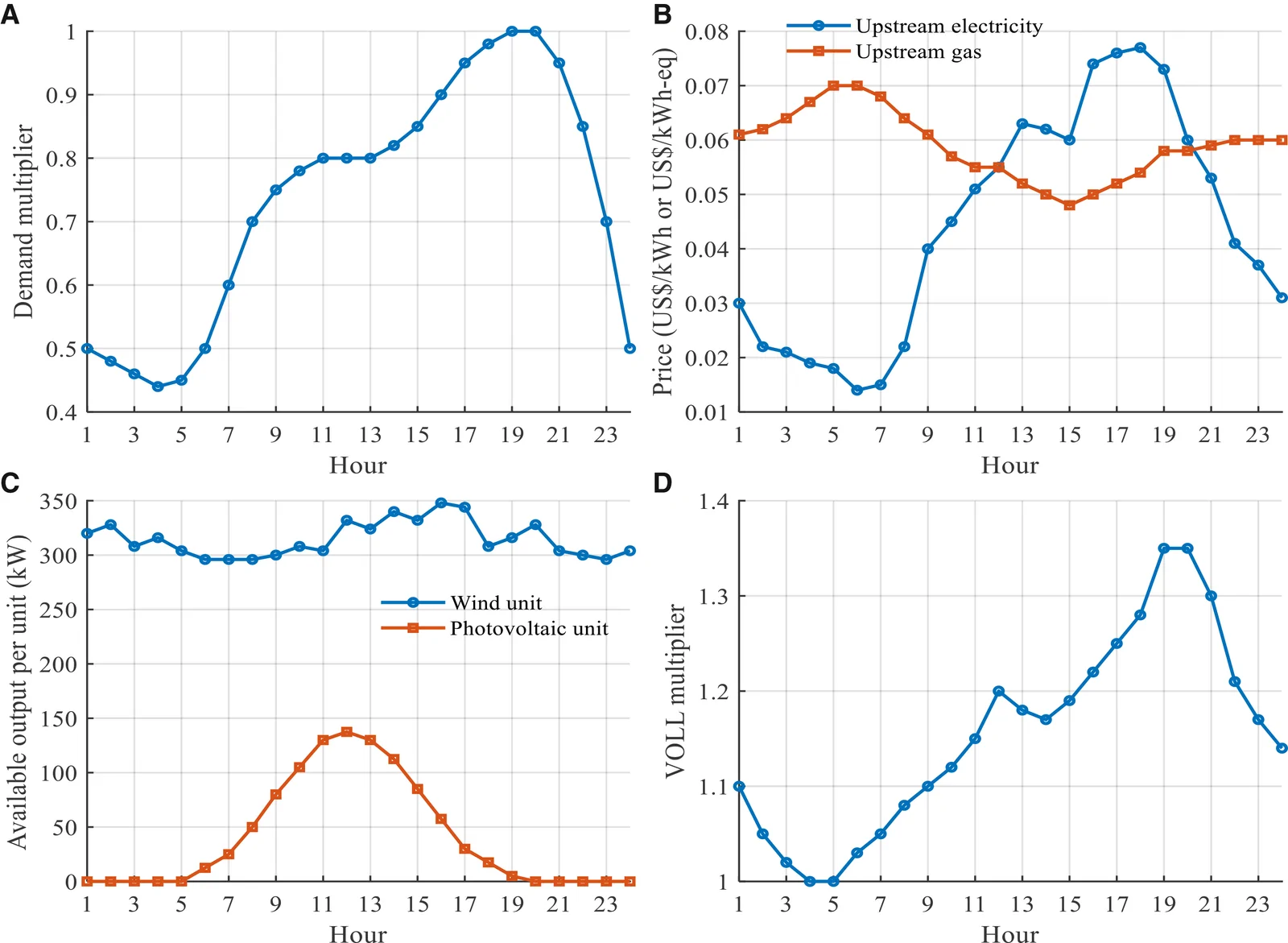
Hourly input data (A) demand multiplier, (B) upstream electricity and gas prices, (C) per-unit wind and photovoltaic output, and (D) value-of-lost-load time multiplier.

Figure 12 summarizes the hourly demand multiplier, upstream electricity and gas prices, renewable availability, and the time multiplier applied to the value of lost load. The final model uses only the p1 demand multiplier. Each wind unit follows the 400-kW profile and each photovoltaic unit follows the 250-kW profile shown in the figure.

The following table summarizes the economic coefficients, uncertainty settings, and numerical parameters used in the simulations.

#### Economic, uncertainty, and numerical settings

**Table:** 

Quantity	Symbol	Value	Unit/note
Controllable generation	*c*^G^	0.05	US$/kWh
Generator start/stop	*C*^su,G^,*C*^sd,G^	0.10/0.10	US$
CHP electrical operation	*c*^CHP^	0.06	US$/kWh
CHP start/stop	*C*^su,CHP^,*C*^sd,CHP^	0.12/0.12	US$
Fuel-cell O&M	*c*^FC,OM^	0.03	US$/kWh
Export-price ratio	*ρ*^sell^	0.80	fraction of purchase price
Renewable curtailment	*c*^curt^	0	US$/kWh
Flow regularization	*c*^flow^	1 × 10^−6^	US$/kW
ESS throughput	*c*^*deg*^	0.005	US$/kWh
Electrical shifting	*c*^DR,E^	0.002	US$/kWh
Thermal shifting	*c*^DR,T^	0.001	US$/kWh
Uncertainty ratio	*β*	0.60	–
Uncertainty budget	*Γ*	2.40	–
Time interval	Δ*t*	1	h
Primary MIP tolerance	–	1 × 10^−6^	relative gap
Primary time limit	–	1800	s
Out-of-sample realizations	–	40	20 in-set and 20 outside-set
Aggregate loss-error tolerance	–	1.0	%
Pointwise loss-error tolerance	–	1.0	kW
Load-shedding reporting tolerance	*ε*_*P*_	0.05	kW
Bus-level EENS reporting tolerance	–	0.05	kWh
Full-service RI threshold	*RI*^*full*^	99.999	%
Adverse-coordinate update tolerance	–	1 × 10^−6^	maximum coordinate change
Loss-reference convergence tolerance	–	0.50	kW
In-set cost-dominance tolerance	*ε*_*C*_	0.10	US$
In-set EENS-dominance tolerance	*ε*_*E*_	0.05	kWh
OOS random seed	–	26072026	common across cases

The bus-dependent base value-of-lost-load coefficients are 0.15, 0.25, and 0.35 US$/kWh. The 0.25-US$/kWh group contains the four hub buses, and the 0.35-US$/kWh group contains buses 17–18, 21–22, 25, and 31–33; the remaining demand buses use 0.15 US$/kWh. These base coefficients are multiplied by the hourly factor in Figure 12D. The models are implemented in GAMS 24.1.2 and solved with CPLEX.

### Quantification and statistical analysis

No inferential statistical tests were applied because the reported values are outputs of deterministic optimization and post-processing calculations. Case comparisons use operating cost, EENS, resilience indices, interruption and recovery metrics, and network-loss measures computed from the optimized schedules. Event-based averages are descriptive equal-weight summaries rather than probability-weighted expected values. Sensitivity analyses vary the prescribed event duration, uncertainty budget, voltage limits, and flexibility costs, while fixed-plan in-set and outside-set stress realizations are used to assess the stability of the comparative ranking. Solver status, relative optimality gap, solution time, and numerical feasibility checks are reported directly from the final GAMS and CPLEX runs.
